# Hippocampal mitochondrial dysfunction and psychiatric-relevant behavioral deficits in spinocerebellar ataxia 1 mouse model

**DOI:** 10.1038/s41598-020-62308-0

**Published:** 2020-03-25

**Authors:** Filip Tichanek, Martina Salomova, Jan Jedlicka, Jitka Kuncova, Pavel Pitule, Tereza Macanova, Zuzana Petrankova, Zdenek Tuma, Jan Cendelin

**Affiliations:** 1 Department of Pathological Physiology, Faculty of Medicine in Pilsen, Charles University, Pilsen, Czechia; 2 Laboratory of Neurodegenerative Disorders, Biomedical Center, Faculty of Medicine in Pilsen, Charles University, Pilsen, Czechia; 3 Department of Physiology, Faculty of Medicine in Pilsen, Charles University, Pilsen, Czechia; 4 Mitochondrial Laboratory, Biomedical Center, Faculty of Medicine in Pilsen, Charles University, Pilsen, Czechia; 5 Laboratory of Tumor Biology, Biomedical Center, Faculty of Medicine in Pilsen, Charles University, Pilsen, Czechia; 6 Department of Biology, Faculty of Medicine in Pilsen, Charles University, Pilsen, Czechia; 7 Laboratory of Proteomics, Biomedical Center, Faculty of Medicine in Pilsen, Charles University, Pilsen, Czechia

**Keywords:** Depression, Spinocerebellar ataxia

## Abstract

Spinocerebellar ataxia 1 (SCA1) is a devastating neurodegenerative disease associated with cerebellar degeneration and motor deficits. However, many patients also exhibit neuropsychiatric impairments such as depression and apathy; nevertheless, the existence of a causal link between the psychiatric symptoms and SCA1 neuropathology remains controversial. This study aimed to explore behavioral deficits in a knock-in mouse SCA1 (SCA1^154Q/2Q^) model and to identify the underlying neuropathology. We found that the SCA1 mice exhibit previously undescribed behavioral impairments such as increased anxiety- and depressive-like behavior and reduced prepulse inhibition and cognitive flexibility. Surprisingly, non-motor deficits characterize the early SCA1 stage in mice better than does ataxia. Moreover, the SCA1 mice exhibit significant hippocampal atrophy with decreased plasticity-related markers and markedly impaired neurogenesis. Interestingly, the hippocampal atrophy commences earlier than the cerebellar degeneration and directly reflects the individual severity of some of the behavioral deficits. Finally, mitochondrial respirometry suggests profound mitochondrial dysfunction in the hippocampus, but not in the cerebellum of the young SCA1 mice. These findings imply the essential role of hippocampal impairments, associated with profound mitochondrial dysfunction, in SCA1 behavioral deficits. Moreover, they underline the view of SCA1 as a complex neurodegenerative disease and suggest new avenues in the search for novel SCA1 therapies.

## Introduction

Spinocerebellar ataxia type 1 (SCA1) is a lethal dominantly-inherited neurodegenerative disease, caused by CAG repeat expansion (> 40 CAG repeats) in the ataxin-1 encoding gene (ATXN1)^1^. This mutation results in ataxin-1 protein toxicity and aggregation which leads, in particular, to cerebellar and brainstem degeneration^2^, although ATXN1 is widely expressed throughout the brain^1^.

SCA1 symptoms usually appear in early middle-age and include motor incoordination and gait deficits followed by muscular and swallowing problems in the later stages of the disease^3^. However, in a similar way to other types of spinocerebellar ataxias (SCAs), over 50% of patients also demonstrate neuropsychiatric issues^4–7^ including cognitive impairments, anxiety, apathy and depression^7–9^. Interestingly, in contrast to progressive ataxia, the psychiatric impairments tend to remain relatively stable over time^8^. Although they are often overlooked, they profoundly impact the quality of life and health outcomes of patients with SCA1 and related diseases^9^. However, the question of whether these psychiatric impairments are causally linked to SCA1 neuropathology, or if they represent an emotional response to SCA1 diagnosis and subsequent physical disability, remains controversial^9^.

Non-motor impairments have also been reported in a mouse SCA1 model with 154 CAG repeats within the endogenous ATXN1 locus (SCA1^154Q/2Q^)^10^. In addition to cerebellar pathology and motor deficits, the SCA1^154Q/2Q^ mice exhibit learning impairments and an alteration to hippocampal plasticity-related functions such as synaptic dynamic disruption, synaptic loss and impaired neural progenitor cell proliferation^11–14^. However, it is not clear whether the hippocampal dysfunctions are directly linked to the SCA1 non-motor symptoms. Moreover, despite the high prevalence of affective symptoms in SCA1 patients^8,9^, analogous behavioral deficits have not been reported to date with respect to any SCA animal models.

Finally, the hippocampal plasticity functions that are impaired in the SCA1^154Q/2Q^ mice crucially depend on proper mitochondrial functioning^15–17^. Mitochondrial functions have been found to be impaired in neurodegenerative diseases^17–19^ and dampened in psychiatric disorders^20–22^. Although recent findings have highlighted the role of mitochondrial dysfunctions in the cerebellar pathophysiology of SCA1^23–25^ and that ATXN1 plays a role in mitochondrial bioenergetics^26^, no examination of non-cerebellar mitochondrial functions in SCA1 has been conducted to date.

This study identifies psychiatric-relevant behavioral deficits in a *knock-in* mouse SCA1 (*SCA1*^*154Q/2Q*^) model. We demonstrate that these deficits precede substantial ataxia and further suggest hippocampal impairments, associated with profound mitochondrial dysfunction, as the underlying neuropathology thereof.

## Results

### Psychiatric-relevant behavioral deficits in the SCA1 mice

**Figure 1:**
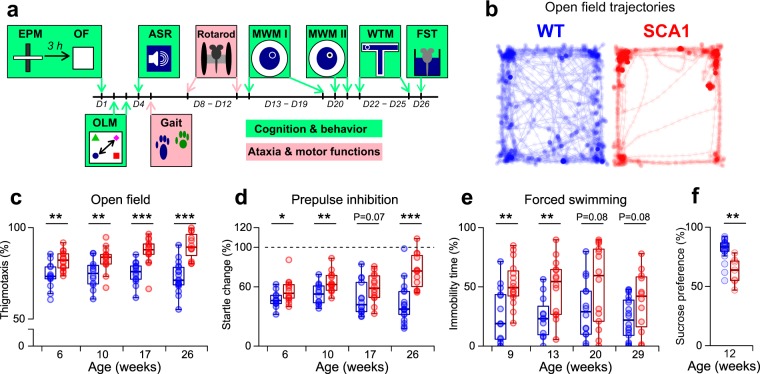
Psychiatric-relevant behavioral deficits in the SCA1 mice. (**a**) Design of the behavioral characterization experiments. D1 indicates the starting day of the experiment (i.e. 6, 10, 17 or 26 weeks of age). It includes the elevated plus maze test (EPM), the open field test (OF), object-location-memory test (OLM), the acoustic startle response and prepulse inhibition (ASR), the gait analysis, the rotarod test, the Morris water-maze test with the hidden (MWM I) and visually marked (MWM II) platform, the water T-maze test (WTM) and the forced swimming test (FST). See Methods for the numbers of animals in each of the experimental groups. (**b**) Representative OF trajectories for the WT and SCA1 mice (aged 6 weeks). (**c**) Thigmotaxis in the OF (i.e. relative distance <5 cm from the arena walls), indicating anxiety-like behavior. (**d**) Relative change in the startle response amplitude following a prepulse stimulus, reflecting prepulse inhibition. (**e**) Relative immobility time during the FST, indicating depressive-like behavior. (**f**) Relative 1% sucrose consumption during the sucrose preference test, indicating depressive-like behavior (N = 21 [WT] and 7 [SCA1] animals). Box-whisker plots indicating the inter-quartile (IQ) intervals (box), 1.5* IQ range (whiskers) and medians (middle line). Each point = 1 animal (**c**–**f**). *P < 0.05, **P < 0.01, ***P < 0.001. The statistical significances are based on the permutational t-test. See Suppl. Table S1 for the detailed results and Suppl. Data S1 for raw data.

In order to investigate functional impairments in *knock-in* SCA1^154Q/2Q^ mice (hereinafter SCA1 mice) compared to healthy littermates (SCA1^2Q/2Q^, hereinafter WT mice), we performed a complex behavioral and motor characterization across four age cohorts (age of entrance to the experiment: 6, 10, 17 and 26 weeks; Fig. 1a). All the assessed univariate indicators obtained from the characterization experiments are listed in Suppl. Table S1 and all the related raw data are provided in Suppl. Data S1.

The SCA1 mice exhibited abnormal behavior in the open field test (OF; Fig. 1b). Specifically, the SCA1 mice were less active than the WT mice (Suppl. Fig. S1) and showed a higher preference to move along the walls (higher thigmotaxis) with respect to all the age cohorts (Fig. 1c). Although the thigmotaxis partially correlated with the distance moved (Suppl. Fig. S1), it remained significant even after adjustment for the distance moved (*OF adj. thigmotaxis*, Suppl. Table S1) and 25% of the most mobile SCA1 mice (distance moved: 40.2–68.71 m) still evinced profoundly higher thigmotaxis than the comparably active WT mice (Suppl. Fig. S1). Similarly, the SCA1 mice exhibited the more frequent avoidance of the open arms during the elevated plus maze test (EPM), although the effect was statistically significant only at the ages of 10 and 17 weeks (Suppl. Fig. S1). Overall, the results obtained from the OF and EPM indicates increased anxiety-like behavior in the SCA1 mice.

The SCA1 mice also exhibited the reduced prepulse inhibition of the startle response with respect to three of the four age cohorts studied (Fig. 1d), suggesting impaired sensorimotor gating. In addition, the older cohorts (17 and 26 weeks of age) evinced a reduced startle amplitude and the prolonged latency of the startle response (Suppl. Fig. S1).

Finally, the SCA1 mice exhibited a significantly higher immobility time during the forced swimming test (FST) concerning the young age cohorts (9 and 13 weeks of age; Fig. 1e). The duration of increased immobility was most pronounced in the first half (0–3 minutes) of the test, at which time the differences between the SCA1 and WT mice attained statistical significance across all the age cohorts (Suppl. Table S1). In order to investigate whether the increased depressive-like behavior was induced by the previous tests, a new cohort of mice (11 animals per group, 6 weeks of age) were subjected to the FST only. The results confirmed that the SCA1 mice were significantly more immobile than the WT mice, specifically during the first half of the test (Suppl. Fig. S1). In order to assess anhedonia-like emotionality, a further cohort of mice (12 weeks of age; N = 21 WT and 7 SCA1 mice) were exposed to the sucrose preference test only. As with the FST results, the SCA1 mice evinced a lower sucrose preference (Fig. 1f), thus confirming substantially enhanced depressive-like behavior in the SCA1 mice, particularly at younger ages (≤14 weeks).

### Altered behavior and learning during the cognitive tasks in the SCA1 mice

**Figure 2:**
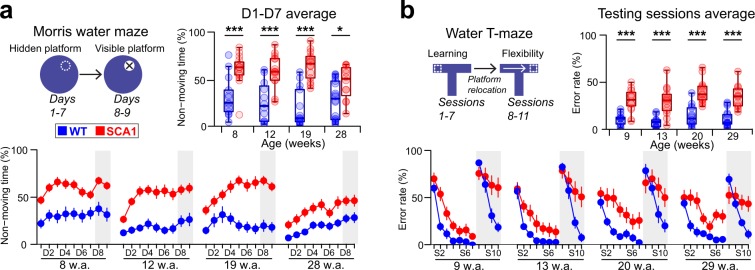
Altered behavior and learning during the cognitive tasks in the SCA1 mice. (**a**) Relative non-moving time during the MWM, averaged over the first 7 days (D1-D7) of the experiment (top) and shown per each session specifically (below). (**b**) Error rate during the water T-maze test, averaged over all the testing sessions (S3-S7 and S10-S11) of the experiment (top) and shown per each session specifically (below). The gray areas in the day/session-specific plots show the visible platform phase of the MWM (**a**) or the reversal phase of the water T-maze test (**b**). The Box-whisker plots (top) indicate the inter-quartile (IQ) intervals (box), 1.5*IQ range (whiskers) and medians (middle lines). Each point = 1 animal. The day/session-specific plots (below) show the means ± SEMs. *P < 0.05, ***P < 0.001. The statistical significances are based on the permutational t-test. w.a. = age in weeks. See Suppl. Tables S5–S8 for the detailed results. See Methods for the numbers of animals in each of the experimental groups.

The object-location memory test did not reveal any genotype-related difference in terms of the exploration of newly-replaced objects (Suppl. Table S2). However, the SCA1 mice needed a longer time to reach both the hidden and visible platforms in the Morris water maze (MWM) (Suppl. Fig. S2, Suppl. Tables S3 and S4). Moreover, the SCA1 mice evinced a strong tendency to non-moving behavior during the MWM (Fig. 2a, Suppl. Tables S5 and S6). Whereas the genotype-related difference in the latency to find the hidden platform was minimal with respect to the young mice (8 and 12 week of age) at the commencement of the MWM (Suppl. Fig. S2 and Table S4), the tendency to non-moving behavior was profoundly higher in the SCA1 mice from the 1st day of testing (Fig. 2a, Suppl. Table S6). This suggests that non-moving behavior is not a secondary consequence of poor learning skills and the increased latency to find the platform in the MWM could not be interpreted as a cognitive deficit in the SCA1 mice. However, the SCA1 mice also learned more slowly to navigate toward the hidden platform in the water T-maze and demonstrated a profound inability to re-learn once the platform had been relocated to the opposite side (Fig. 2b, Suppl. Tables S7 and S8), thus suggesting substantially reduced memory and cognitive flexibility.

### Non-motor deficits characterize the early SCA1 stage in mice better than does ataxia

**Figure 3:**
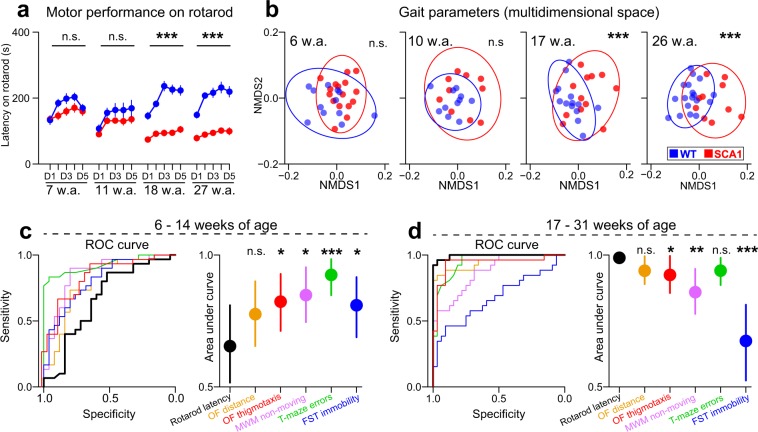
Non-motor deficits characterize the early SCA1 stage in mice better than does ataxia. (**a**) Latency on the accelerating rotarod over 5 days (D) of experimentation. The points are means ± SEMs. The P-values are based on comparing the averaged values from the whole experiment via the permutational t-test. See Suppl. Tables S9–S11 for the detailed results. (**b**) Visualization of the dissimilarities in the gait parameters using non-metric multidimensional scaling (NMDS). The P-values are based on the permutational multivariate analysis of variance (PERMANOVA). The ellipses show the 90% confidence intervals for point occurrence (each point = 1 animal). (**c**,**d**) ROC curve and area under the ROC curve showing the ability of the sensitive indicators to correctly classify the mice into genotypes. The vertical lines imply 95% confidence intervals. The significances are based on bootstrapping and indicate the difference between the AUC of a given indicator versus the AUC of rotarod latency. *P < 0.05, **P < 0.01, ***P < 0.001. n.s. = not significant. w.a. = age in weeks. OF = open field. MWM = Morris water maze. FST = forced swimming test. See Methods for numbers of animals per each experimental group.

Although the older age cohort (18 and 27 weeks of age) SCA1 mice exhibited substantial motor impairments, the younger mice (7 and 11 weeks of age) performed only mildly or insignificantly worse on the accelerating rotarod than did the WT mice (Fig. 3a; Suppl. Tables S9 and S10). Similarly, the older SCA1 mice (17 and 26 weeks of age) had substantially abnormal gaits, whereas the younger SCA1 mice (6 and 10 weeks of age) evinced no significant gait dissimilarities from the controls (Fig. 3b, Suppl. Table S11).

Of all the indicators assessed in the characterization experiments (Fig. 1a, Suppl. Table S1), we selected those with the highest sensitivity to the genotype (*sensitive indicators*; Suppl. Methods). We identified the following *sensitive indicators*: distance moved in the OF, thigmotaxis in the OF, averaged rotarod latency, non-moving behavior in the MWM (average from D1-D7), error rate in the water T-maze (average from S2-S7 and S10-S11) and immobility time in the FST.

Generally, the *sensitive indicators* differed significantly between *young* (aged 6–14 weeks) and *old* (aged 17–31 weeks) SCA1 mice (Suppl. Table S12; Suppl. Fig. S3). Since the functional impairment patterns did not differ significantly within these two groups (Suppl. Table S12), the data were merged into two datasets only (*young* and *old* mice) for further analyses. Since the *young* SCA1 mice did not exhibit profound ataxia, potentially confounding behavioral results, we devoted greater attention to these age cohorts when analyzing the behavior and underlying neuropathology of the SCA1 mice.

In order to identify the behavioral measures which might be affected by even subtle signs of ataxia in the *young* SCA1 mice, we examined whether the *sensitive indicators* correlated with decreased rotarod performance and/or abnormal gait (expressed via the *principal components* from the 4 most sensitive gait parameters; Suppl. Table S1; Suppl. Methods). Gait and rotarod performance were inter-correlated and gait showed its effect on the distance moved in the OF (Suppl. Table S13), suggesting that the activity in the OF might be partially motor-dependent in young SCA1 mice. Although MWM non-moving was not significantly associated with ataxia, it correlated with distance moved in the OF, suggesting that this measure may reflect general inactivity (Suppl. Table S13).

In order to test whether the non-motor *sensitive indicators* characterize the early SCA1 mice significantly better than do their motor functions, we constructed a ROC curve that visualizes and quantifies the ability of the *sensitive indicators* to successfully classify the mice into genotypes (WT vs. SCA1). All the non-motor *sensitive indicators* distinguished the SCA1 mice from the WT mice more accurately than did rotarod latency at younger ages (Fig. 3c). On the other hand, as the ataxia rapidly worsened in the older mice (≥17 weeks of age), rotarod latency differentiated the mice more accurately than did most of behavioral deficits after 17 weeks of age (Fig. 3d). Taken together, the results suggest that the early SCA1 stage in the mouse model is characterized particularly by non-motor behavioral and cognitive deficits.

### Hippocampal atrophy precedes cerebellar degeneration and reflects the severity of behavioral deficits

**Figure 4:**
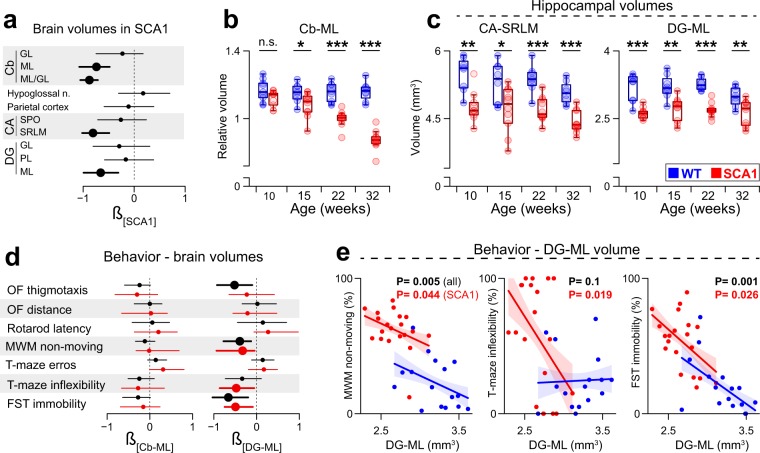
Hippocampal atrophy precedes cerebellar degeneration and reflects the severity of the behavioral deficits. (**a**) Standardized regression coefficients (*β*) and 95% confidence intervals (95% CI) for the effect of the SCA1 genotype on brain volumes. The thickness indicates that 95% CI did not cross zero. Cb = cerebellum. CA = *Cornu ammonis*. DG = dentate gyrus. GL = granular layer. ML = molecular layer. SPO = stratum pyramidale and oriens. SRLM = stratum radiatum and lacunosum-moleculare. PL = polymorph layer. N = 8 animals per group (32 weeks of age). See Suppl. Table S14 for details. (**b**) Cerebellar molecular/granular layer volume ratio (Cb-ML). (**c**) CA-SRLM and DG-ML hippocampal volumes. Box-whisker plots (**b**,**c**) indicating inter-quartile (IQ) intervals (box), 1.5*IQ range (whiskers) and medians (middle line). Each point = 1 animal. N (**b**,**c**) = 8 (WT) and 10 (SCA1) animals (9 per group in case of the youngest cohort). *P < 0.05, **P < 0.01, ***P < 0.001. n.s. = not significant. The P-values are based on the permutational t-test (**b**,**c**). (**d**) *β* for the effect of the Cb-ML (left) and DG-ML (right) volumes on the *sensitive indicators* and 95% CI. The thickness indicates that 95% CI did not cross zero. The black lines show the results of the models that included both the WT and SCA1 mice with the genotype as a covariate, whereas the red lines show the results obtained from the SCA1 mice-specific data (Suppl. Tables S17–S24). (**e**) Relationship between the DG-ML volumes and the *sensitive indicators* significantly associated with the DG-ML in the SCA1 mice: relative non-moving time during the MWM (D1-D7 average; left), error rate during the flexible phase of the water T-maze test (middle) and the immobility time during the FST (right). The lines indicate model fits and their standard errors. The P-values are based on percentile bootstrapping (Suppl. Tables S20 and S21). Each point = 1 animal. N = 17 (WT) and 19 (SCA1) animals (aged 6–15 weeks; **d**,**e**).

In order to explore region-specific brain volumetric atrophy in the SCA1 mice (Suppl. Fig. S4, Suppl. Data S1), we performed brain volumetry analyses via the histological examination of the mouse brains from the behavioral characterization experiments. Preliminary analysis based on the oldest age cohort (the mice euthanized at 32 weeks of age; 8 animals per group) identified the molecular layer of the cerebellum (its relative volume per granular layer volume [Cb-ML]) and two regions of the hippocampus (the dentate gyrus molecular layer [DG-ML] and the inner layers of the *Cornu ammonis* [stratum radiatum + lacunosum-moleculare; CA-SRLM]) as those regions of the brain most sensitive to volumetric atrophy in the SCA1 mice (Fig. 4a, Suppl. Table S14). Thus, these brain regions were assessed across all the age cohorts (N = 8 WT and 10 SCA1 mice per age cohort; 9 per group in the youngest cohort).

The relative Cb-ML volume gradually decreased as the disease progressed in the SCA1 mice. Whereas it was found to be only insignificantly or mildly reduced in the young age cohorts (aged ≤ 15 weeks), it was seen to have decreased dramatically in the older SCA1 mice (Fig. 4b).

In contrast to the Cb-ML volumes, the hippocampal volumes (both CA-SRLM and DG-ML) were found to be significantly reduced in the SCA1 mice across all the age cohorts (Fig. 4c) and, thus, preceded the significant Cb-ML atrophy. However, the SCA1 mice also evinced reduced overall brain weight with respect to all the age cohorts (P < 0.05), and the hippocampal volumes partially depended on the overall brain weight (Suppl. Tables S15 and S16). When the total brain weight was taken into account (included as a covariate in the general linear model along with the genotype effect), the significant genotype effect disappeared with concern to the CA-SRLM in 1 of the age cohorts (Suppl. Table S15, Suppl. Fig. S4). However, with respect to the DG-ML volume, the volumetric reduction in the SCA1 mice remained significant for all the age cohorts (Suppl. Table S16, Suppl. Fig. 4), thus implying that the DG-ML atrophy in the SCA1 mice is not simply a consequence of overall brain tissue loss.

We subsequently investigated the association between the region-specific volumes and the individual severity of the functional impairments (the *sensitive parameters*), specifically concerning the *young* (aged ≤ 15 weeks) SCA1 mice. The Cb-ML and CA-SRLM volumes and total brain weight were not found to be associated with any of the functional impairments at the statistically significant level, although an apparent trend was evident toward a positive association between the error rate in the water T-maze test and the Cb-ML volume (Fig. 4d; Suppl. Tables S17–S19). When we repeated the same analysis also for absolute volume of the Cb-ML, we found that it was significantly associated only with rotarod latency (Suppl. Table S17). In contrast, DG-ML atrophy was associated with an increased non-moving behavior in the MWM, more severe cognitive flexibility impairments in the water T-maze test and increased depressive-like behavior during the FST (Fig. 4d,e, Suppl. Table S20).

The association between non-motivated/despaired behavior (with respect to both MWM and FST) and the DG-ML volume was not found to be specific to the SCA1 mice, since including the WT mice into the analysis even strengthened the association (Suppl. Table S21). In contrast, the association of the DG-ML volume with cognitive flexibility in the water T-maze test was seen to be specific only to the SCA1 mice since it disappeared once the WT mice were included. In a similar way to the SCA1-specific brain behavior association, the Cb-ML, CA-SRLM and brain weight were not significantly associated with any of the *sensitive indicators* when all the animals (WT and SCA1) were included (Suppl. Tables S22–S24).

In conclusion, the brain volumetry data indicate hippocampal, particularly DG-ML, volumetric atrophy that is directly linked to the individual severity of some of the behavioral deficits observed in the young SCA1 mice.

### Impaired hippocampal neurogenesis in the SCA1 mice

**Figure 5:**
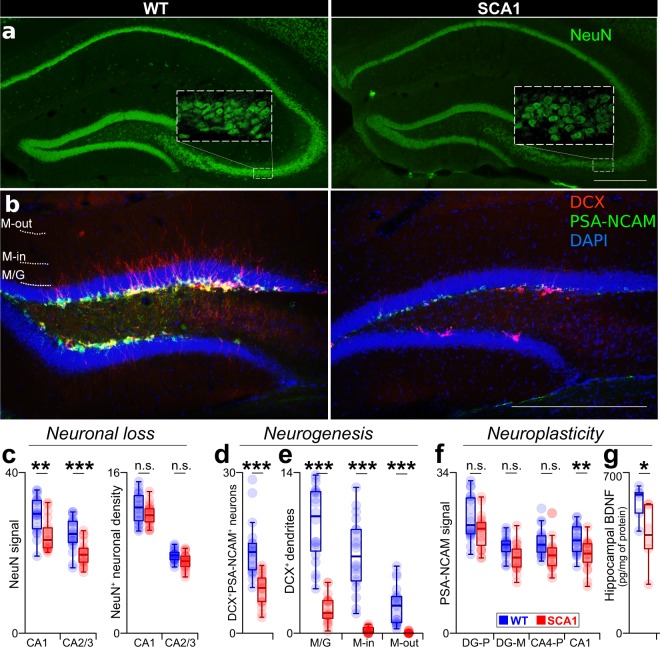
Impaired hippocampal neurogenesis in the SCA1 mice. (**a**) Representative images of hippocampal sections stained for NeuN. Scale bars (**a**,**b**) = 300 µm. (**b**) Representative images of the DG double stained for DCX and PSA-NCAM (with DAPI). (**c**) NeuN immunofluorescence intensity in the CA1 (left) and CA2/3 (middle-left) pyramidal layers and the density of NeuN+ neurons (per 1,000 µm^2^) in the same hippocampal regions (right). (**d**) Density of DCX^+^PSA-NCAM^+^ neurons per 100 µm of the DG subgranular zone. (**e**) Density of DCX^+^ dendrites crossing the line on the border of the DG-G and DG-ML (M/G; left), inner part of the DG-ML (M-in; middle) and outer part of the DG-ML (M-out; right), per 100 µm. (**f**) PSA-NCAM immunofluorescence in the DG polymorph layer (left; DG-P), DG-ML (middle-left), CA4 pyramidal (middle-right; CA4-P) and CA1 stratum lacunosum-moleculare (right; CA1) hippocampal layers. N (**c**-**f**) = 5 animals per group (with 4 sections per animal). Each point = 1 section. The statistical significances were based on the permutational linear mixed-effect models (See Suppl. Tables S25 and S26 for the detailed results) using 4 replicates per animal and with the animal identity representing the random-effect factor. (**g**) Hippocampal BDNF level (pg/mg of proteins). N = 6 animals per group. P = 0.046 (permutational t-test). Each point = 1 animal. Box-whisker plots (**c**–**g**) indicating the inter-quartile (IQ) intervals (box), 1.5*IQ range (whiskers) and medians (middle line). *P < 0.05, **P < 0.01, ***P < 0.001. n.s. = not significant.

In order to evaluate whether decreased neuronal density contributes to volumetric hippocampal atrophy, we performed the immunofluorescence staining of CA mature (NeuN^+^) pyramidal neurons and immature (DCX^+^PSA-NCAM^+^) neurons in the DG (sub)granular layer (N = 5 per group; 13–15 weeks of age). The SCA1 mice exhibited a significant reduction in NeuN immunofluorescence in both the evaluated subregions (CA1 and CA2/3). However, the density of the NeuN^+^ neurons evinced only an insignificant trend toward decreased neuronal densities in the SCA1 mice (Fig. 5a,c, Suppl. Table S25), thus suggesting decreased NeuN expression/antigenicity without marked neuronal loss (Fig. 5a,c). Interestingly, the SCA1 mice also evinced distinctly lower numbers of immature (DCX^+^ PSA-NCAM^+^) neurons in the DG subgranular layer (Fig. 5b,d). In addition, the dendrites of the immature (DCX^+^) neurons were dramatically impoverished and were practically absent in the outer parts of the DG-ML in the SCA1 mice (Fig. 5b,e). These results thus suggest that profoundly diminished neurogenesis accompanies hippocampal volumetric atrophy in the SCA1 mice.

Since the PSA-NCAM fluorescence of the non-neuronal morphology indicates the occurrence of neuroplasticity processes, such and neurite growth and formation of synapses^27–30^, we measured the PSA-NCAM immunofluorescence in four hippocampal areas: the DG hilus, DG-ML, CA4 pyramidal and CA1 stratum lacunosum-moleculare (CA1-SLM) layers. We found that fluorescence was reduced in one (the CA1-SLM) but not in the other hippocampal areas (Fig. 5f), suggesting that reduced neuroplasticity processes may also partially contribute to the hippocampal atrophy in the SCA1 mice.

Since neuronal survival, neurogenesis and neurite growth depend on sufficient levels of neurotrophic factors, we subsequently employed ELISA to measure the hippocampal levels of *brain-derived neurotrophic factor* (BDNF) as a pivotal representative of the neurotrophic factor class. The hippocampal BDNF level was reduced in the SCA1 mice (Fig. 5g) which suggests the possible role of reduced BDNF levels in the afore-mentioned hippocampal impairments observed in these mice.

### Normal urinary corticosterone level in the SCA1 mice

**Figure 6:**
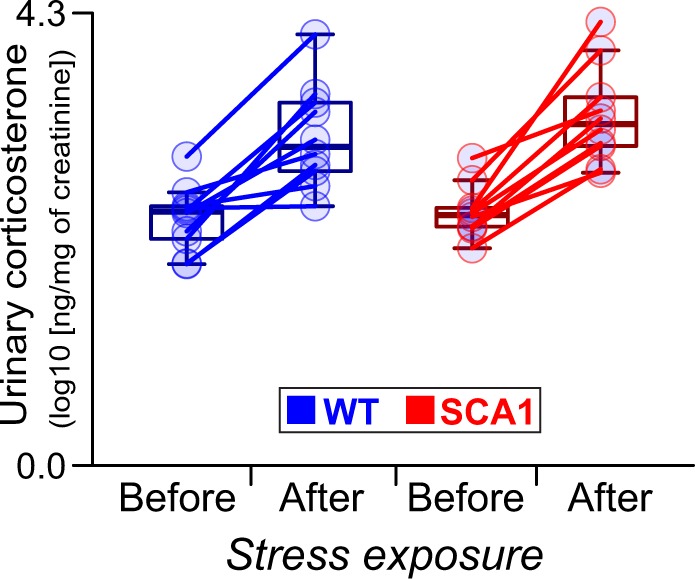
Normal urinary corticosterone level in the SCA1 mice. Urinary corticosterone (per creatinine) before and 1 hour after acute stress. Results of the permutational linear mixed-effect model: *stress* (within-subject factor): F_1,18_ = 79, P < 0.001; *genotype*: F_1,18_ = 0.78, P = 0.4; stress*genotype interaction: F_1,18_ = 0.48, P = 0.5. N = 9 animals per group. Box-whisker plots indicating the inter-quartile (IQ) intervals (box), 1.5*IQ range (whiskers) and medians (middle line). The lines connect points representing values from 1 animal.

In order to test whether the increased stress response and the consequent increase in the corticosterone concentration contribute to the hippocampal impairments and abnormal behavior or the correlation thereof, we measured urinary corticosterone at the basal state and 1 hour following the initiation of stress (9 mice per group; aged 10 weeks). While the corticosterone concentration (standardized per creatinine) was affected by the acute *stress* (within-subject factor; F_1,18_ = 79, P < 0.001), it was not significantly influenced by the *genotype* (F_1,18_ = 0.78, P = 0.4) nor the stress*genotype interaction (F_1,18_ = 0.48, P = 0.5; Fig. 6).

### Hippocampal-specific mitochondrial dysfunction in the young SCA1 mice

**Figure 7:**
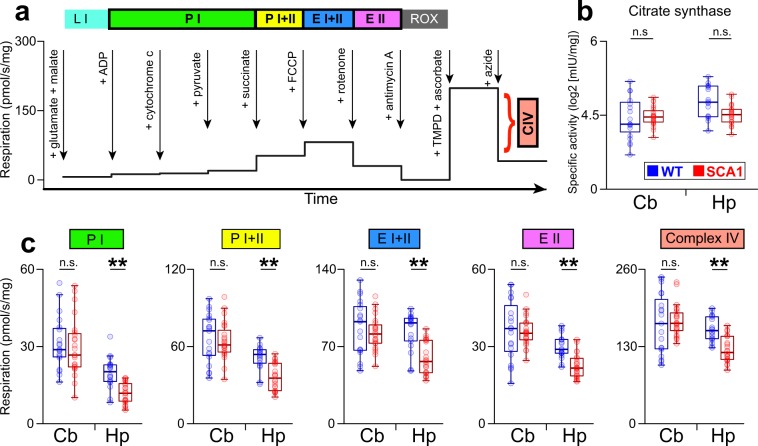
Hippocampal-specific mitochondrial dysfunction in the young SCA1 mice. (**a**) Design of a substrate-uncoupler-inhibitor titration protocol for measuring the mitochondrial respiratory capacity. (**b**) Specific enzymatic activity of citrate synthase in the cerebellar and hippocampal tissues (log 2 [mIU/mg of tissue]). (**c**) Mitochondrial respiration (pmol O_2_/s/mg of homogenized tissue) in different states of the substrate-uncoupler-inhibitor protocol, reflecting complex I OXPHOS capacity in the ADP-activated state of oxidative phosphorylation (*P I*), complex I + II OXPHOS capacity (*P I* + *II*), maximum capacity for electron transport (*E I* + *II*), complex II uncoupled capacity (*E II*) and *complex IV* capacity, separately for the cerebellum (Cb) and the hippocampus (Hp) and for the WT (N = 5) and SCA1 (N = 6) mice (11–13 weeks of age; 4 samples for each mouse and brain structure). Box-whisker plots (**b**,**c**) indicating the inter-quartile (IQ) intervals (box), 1.5*IQ range (whiskers) and medians (middle lines). Each point = 1 sample. *P < 0.05, **P < 0.01, ***P < 0.001. n.s. = not significant. P-values were based on the permutational linear mixed-effect models (Suppl. Tables S27) using 4 samples per animal and with the animal identity representing the random-effect factor. See Suppl. Methods for the abbreviations.

In order to investigate whether the brain abnormalities and behavioral deficits identified might be related to mitochondrial dysfunction, we employed high-resolution respirometry to measure mitochondrial respiration in the cerebellar and the hippocampal tissue of the SCA1 mice and their healthy littermates (N = 6 SCA1 and 5 WT mice; aged 11–13) applying the substrate-uncoupler-inhibitor titration (SUIT) protocol^31^ (Fig. 7a) and citrate synthase activity measurements (Suppl. Methods).

The SCA1 mice did not exhibit any statistically significant differences compared to their WT counterparts in terms of citrate synthase activity although an apparent trend was seen toward a decreased citrate synthase activity in hippocampus of the SCA1 mice (Fig. 7b, Suppl. Table S27). However, the SCA1 mice did suffer from a substantial reduction in mitochondrial respiration, which was surprisingly specific to the hippocampus, i.e. it was not detected in the cerebellum. Interestingly, the hippocampal tissue of the SCA1 mice exhibited compromised respiration in all the evaluated respiratory states, including reduced complex I OXPHOS capacity in the ADP-activated state of oxidative phosphorylation (*P I*), complex I + II OXPHOS capacity (*P I* + *II*), maximum capacity for electron transport (*E I* + *II*), complex II uncoupled capacity (*E II*) and *complex IV* capacity (Fig. 7c, Suppl. Table S27). Moreover, when the citrate synthase activity was taken into account (included as a covariate in the mixed-effects model; Suppl. Methods) the genotype effect on hippocampal respiration remained significant in respect to all respiratory states except for P I (Suppl. Table S28). To validate results of previous study pointing out the reduced complex I/maximal respiration ratio in the cerebellum of the SCA1 mice^23^, we performed the same analysis with negative results (P = 0.44).

These results suggest that a profound hippocampal mitochondrial deficit may provide an essential driver of hippocampal impairment and the related behavioral deficits in SCA1 mice.

## Discussion

The results of the study led to the identification of previously undescribed psychiatrically-relevant behavioral deficits that preceded the substantial ataxia in the SCA1 mice. Although we cannot entirely exclude the possibility that certain subtle signs of physical deterioration (e.g. subtle motor deficits or fatigue) may partially contribute to some of the behavioral abnormalities, our data suggest that at least some of the psychiatrically-relevant behavioral deficits might be independent of physical and motor deterioration. This is in agreement with several clinical studies that have demonstrated that SCA patients often suffer from psychiatric issues such as anxiety, depression and cognitive dysfunction^4–9^. Moreover, such psychiatric issues commonly emerge at the outset of the disease and their severity tends to remain relatively stable over time in contrast to physical disability^5,9^.

Our behavioral findings are in line with previous reports of lower performance in the MWM and fear conditioning in the same mouse^10,12^. However, we suggest that MWM performance is confounded by increased immobility and, therefore, that it does not reflect learning impairments in the SCA1 mice. In contrast to a previous report^10^, but in line with the results of other studies^32,33^, we determined only a marginal and statistically insignificant deterioration in the rotarod performance of the young SCA1 mice. The discrepancy in the results of the various studies considered may stem from the differing designs of the rotarod test. Finally, since we identified highly-sensitive and easily-measurable behavioral deficits in the SCA1 mice, analogous to psychiatric issues in humans, our findings will facilitate the further studies of these impairments and the relevant therapeutic strategies by means of a mouse model.

In a similar way to the behavioral findings, we determined atrophy and functional impairments in the hippocampus (the key brain structure in terms of cognition and emotions^34^) of the SCA1 mice. The reduced numbers of immature neurons along with a dramatic reduction in the dendrites thereof imply hugely impaired neurogenesis in the SCA1 mice corresponding to the reported inhibition of the proliferation of hippocampal progenitor cells^14^. Moreover, the reduced intensity of the PSA-NCAM immunofluorescent signal in one of hippocampal sub-regions suggests that reduced synapses formation and neurite growth may also contribute to the hippocampal atrophy of the SCA1 mice^27–29^. This corresponds to the reported CA3 pyramidal dendrite pathology and altered synaptic formation dynamics in the same mouse model^11,12^. Finally, the reduced NeuN immunofluorescence in the CA pyramidal layer without a significant reduction in the NeuN^+^ neuronal density suggests reduced NeuN expression or antigenicity, which may reflect a diseased neuronal phenotype in the hippocampus of the SCA1 mice^35–38^. Although the insignificant trend towards decreased neuronal density in the SCA1 mice may, hypothetically, indicate the commencement of neuronal loss that had already been reported in the CA2 of older SCA1 mice^39^, this trend might also be explained by the confounding effect of significantly decreased NeuN expression^36^. Taken together, we concluded that the SCA1 mice suffer from hippocampal atrophy from a young age, which is most likely caused by a combination of impaired neuroplasticity processes, dendrite atrophy, suppressed neuroproliferation and markedly impaired neurogenesis.

Although it has been demonstrated that BDNF play a role in cerebellar neuropathology in SCA1^40^, the hippocampal BDNF deficiency that we determined has not been described in any other SCA animal model to date. Since BDNF plays an important role in neurite growth, neuronal survival and neurogenesis^41–43^, we speculate that BDNF deficiency may partially contribute to the hippocampal neuropathology described above and may represent a hopeful target for hippocampal-focused SCA1 therapy.

Furthermore, our results indicate a direct association between the DG-ML volume and the individual severity of certain behavioral deficits, i.e. depressive-like behavior and cognitive inflexibility. Although such a direct association has not previously been described in any other SCA model, the results do correspond to reported DG atrophy in a mouse models of mood disorders^44,45^ and the correlation between DG volumetric atrophy and depression severity/duration in depressed humans^46–48^. Similarly, the association determined between DG-ML atrophy and inflexibility in the SCA1 mice corresponds with the known role of hippocampal neurogenesis, that is related to the DG volume^44^, for cognitive flexibility^49^. A potential explanation for this association is that increased stress leads to both hippocampal atrophy and behavioral deficits. However, the young SCA1 mice evinced normal corticosterone levels and the CA-SRLM volume was not associated with behavioral deficits, despite the high sensitivity of CA to increased levels of corticosterone^44,50^, thus suggesting that increased stress does not mediate hippocampal atrophy and its direct link to the abnormal behavior of the SCA1 mice. In conclusion, our results point to hippocampal impairments as the neuropathology that underlay some of the behavioral deficits seen in the SCA1 mice. This finding offers a potential biological explanation for the occurrence of psychiatric issues in human SCA1 patients. Although the finding must be validated directly via trials involving human patients, it does correspond to the essential role of the hippocampus in human emotion, cognition and psychiatric disorders^34^.

The lack of cerebellar degeneration in the young SCA1 mice, our correlational analysis and the fact that the mice with cerebellar-specific degeneration exhibit a number of opposite abnormalities (lack of immobility in the FST and MWM, reduced thigmotaxis in the OF and higher relative times in the open arms in the EPM)^51–55^ suggest that the behavior of the SCA1 mice may be relatively independent of motor deficits and cerebellar degeneration. On the other hand, the SCA1 mice suffer from cerebellar inflammation prior to the onset of cerebellar degeneration^33^, and cerebellar inflammation is able to induce depressive-like behavior in mice^56^. Thus, we cannot exclude the possibility that the cerebellar inflammation also contributed to non-motor deficits in the SCA1 mice. Moreover, since the cerebellum modulates hippocampal functions and excitability^57–59^, it is possible that the hippocampus itself may be impacted by the cerebellar inflammation.

In addition to hippocampal atrophy and the related behavioral deficits, we also identified corresponding mitochondrial dysfunction in the hippocampus (but not in the cerebellum) of the young SCA1 mice. Although mitochondrial dysfunction comprises one of a number of common pathological mechanisms shared by various neurodegenerative diseases^19^, it has been studied only rarely in spinocerebellar ataxias focusing exclusively on cerebellar tissue/cells^23–25^. The fact that we did not determine any mitochondrial dysfunction in the cerebellum contradicts the results of previous studies^23,24^. This discrepancy could be due to the use of different methods (e.g. mass spectroscopy^24^) or differences in the SCA1 mouse models (Purkinje cell-specific SCA1 model^23^). However, the ATXN1 gene is also richly expressed in the hippocampus and ataxin-1 may play a general role in mitochondrial bioenergetics^26^. Moreover, the plasticity-related functions in the hippocampus, such as neurogenesis and neurite growth, are energetically extremely demanding and crucially depend on proper mitochondrial functioning^16–18,60,61^. Interestingly, hippocampal mitochondria dysfunction rapidly inhibits hippocampal plasticity and leads to hippocampal atrophy and impaired behavior^62^, i.e. the condition observed in the SCA1 mice. These findings therefore suggest that mitochondrial dysfunction in SCA1 extends beyond the cerebellum and may be even more severe in the hippocampus, at least during the early stages of SCA1. In this respect, mitochondrial dysfunctions may constitute an essential driver of plasticity-related hippocampal impairment, subsequent tissue atrophy and abnormal behavior in SCA1.

The results suggest that targeting the mitochondrial function provides a promising approach in terms of enhancing hippocampal-related functions in SCA1. Agents developed to improve certain mitochondrial functions have already been shown to improve cerebellar neuropathology in SCA1 mice^23,24^. Moreover, lithium, a drug that stimulates mitochondrial functions in various brain and mitochondrial diseases^63–66^, has been shown to improve learning and hippocampal neuropathology in SCA1 mice^12^. On the other hand, the mitochondrial function depends on physical activity^67^, that is reduced in SCA1 mice from an early age (independently of hippocampal atrophy), which could lead to a secondary reduction in the mitochondrial function. In this case, forced physical activity would rapidly alleviate some of the deficits. Finally, it would be advisable to validate the role of hippocampal bioenergetics disruption directly on human SCA1 patients and/or a human-induced pluripotent stem cell neuronal culture derived from patients^68,69^.

In conclusion, our results imply the essential role of hippocampal impairments, associated with profound mitochondrial dysfunction, in psychiatric-relevant behavioral deficits in SCA1 mice. Thus, this study supports the view of SCA1 as a complex neurodegenerative disease involving a non-motor disease component independent of progressive ataxia. As the non-motor component severely disturbs subjective well-being and leads to worsened prognoses and poor health outcomes in SCA patients^8,9^, we argue that it deserves increased scientific attention aimed at identifying an effective treatment. We trust that our study will establish some of the groundwork for such research in the future.

## Methods

### Animals

We used B6.129S-Atxn1^tm1Hzo^/J *knock-in* mice (Jackson Laboratory)^10^ for testing purposes. Heterozygous mice with 154 CAG repeats within exon 8 of the targeted endogenous mouse ATXN1 locus (SCA1^154Q/2Q^) were used along with homozygous control mice with normal CAG repeats in both ATXN1 loci (SCA1^2Q/2Q^). For practical reasons, only males were used in the experiments unless specified elsewhere (females were not available at the time in sufficient numbers for the experiments due to their prioritization for breeding purposes). The mice were group-housed (≤5 animals per cage), again unless specified otherwise. The animals were kept under controlled temperature (23  ±  1 °C) and humidity (30–70%) conditions with a 12 h light/dark cycle with food and water available *ad libitum*. All the experiments were conducted in full compliance with European Union Guidelines for Scientific Experimentation on Animals and with the permission of the Ethical Commission of the Faculty of Medicine in Pilsen. All the protocols followed in this study were approved by the Ethical Committee of the Ministry of Education, Youth and Sports of the Czech Republic (approval no. MSMT-10669/2016–4 and MSMT-27476/2016-2) according to the Guide for the Care and Use of Laboratory Animals (Protection of Animals from Cruelty Law - Act No. 246/92, Czech Republic). Every effort was made to minimize suffering.

### Behavioral experiments

The characterization experiments involved the mice (SCA1^154Q/2Q^ and their healthy littermates [SCA1^2Q/2Q^]) undergoing a 4-week-long battery of behavioral tests. The mice entered the experiments in the form of 4 age cohorts: I) 6 weeks (± 4 days), II) 10 weeks (± 6 days), III) 17–18 weeks (± 2 days) and IV) 26–28 weeks. The number of animals per cohort (WT, SCA1) was: I) 13 and 16, II) 12 and 14, III) 14 and 14 and IV) 18 and 12. The design of the behavioral and motor characterization experiment is shown in Fig. 1a and described in detail in Suppl. Methods. With the exception of the 1st day, the mice were subjected to a maximum of 1 test per day. In the subsequent behavioral experiments that were aimed at validating the findings from the basic characterization stage, the mice were subjected to the FST (11 WT and 11 SCA1) at the age of 6 weeks (± 4 days) or the sucrose preference test (21 WT and 7 SCA1) when aged between 10 and 12 weeks. All the tests proceeded during the light period (6 am to 6 pm). All the mice were habituated to contact with the experimental personnel for at least 1 week prior to the commencement of the experiment.

### Histological examinations and immunofluorescence

The mice from the characterization experiments were euthanized (aged 10, 15, 22 or 32 weeks) and transcardially perfused with Ringer’s solution followed by 4% phosphate-buffered paraformaldehyde (PFA, pH 7.4). The brains were subsequently removed and left in PFA for 2 hours and then in sucrose (15, 20, 30%) for 2 days. Finally, they were weighed and frozen at −80 °C; as described in detail elsewhere^70,71^. They were then cut into 40 µm wide frontal slices and stained using Cresyl Violet (Nissl staining) or immunofluorescent methods.

The Nissl-stained slices were scanned using an *Olympus DP70* digital camera coupled to an *Olympus BX51* microscope (*Olympus, Japan*) with a *PlanApo N* 2×/0.08 objective. The volumes were assessed employing the point grid method and the Cavalieri principle using *Gimp* software. The assessed brain regions (Fig. 4a, Suppl. Table S14) include several cerebellar and hippocampal (Suppl. Fig. S4) sub-regions, the hypoglossal nucleus and the thickness of the parietal cortex (Suppl. Fig. S4, Suppl. Methods, Suppl. Data S1). We firstly performed the preliminary analysis of the oldest age cohort (8 animals per group) and, subsequently, those brain regions identified as genotype-sensitive (Fig. 4a) were assessed across all the age cohorts (N = 8 WT and 10 SCA1 mice and 9 per group in the youngest cohort). We used the Allen Brain Atlas (http://atlas.brain-map.org) for orientation in the brain slices.

We performed immunofluorescent staining for the NeuN (mature neurons) and double staining for the DCX and PSA-NCAM (immature neurons and neuroplasticity processes). 2 or 4 brains were stained at once (i.e. in 1 of 3 *blocks*), with equal numbers of WT and SCA1 mice in each of the blocks. All the staining was performed with DAPI (see Suppl. Methods for a list of the antibodies applied and their concentrations). We evaluated 5 animals per group (aged 13–15 weeks) and used every 6th slice for each animal and staining, commencing from the 12^th^ slice containing the hippocampus (from the frontal part); a total of 4 slices per animal. Imaging was performed using a fluorescent *Olympus BX51* microscope and an *Olympus DP70* digital camera (Olympus, Japan). Detailed images were acquired by means of an Olympus IX83 spinning disk confocal microscope (Olympus, Germany). Images were analyzed using *Fiji* software. The NeuN staining was analyzed by means of measuring the color intensity while subtracting the immunofluorescent intensity in neighboring sites without the presence of neuronal bodies (the CA strata oriens; Suppl. Fig. S5) and via the manual counting of the neurons in the pyramidal layers of two hippocampal sub-regions: CA1 and CA2/3. The PSA-NCAM^+^ DCX^+^ neurons were counted manually in at least 250 µm of the DG subgranular zone per 1 evaluated hippocampus (see Suppl. Methods for details). The DCX^+^ neuronal dendrites were quantified by means of the number of crossing lines (at least 250 µm per evaluated DG) located in three positions: i) the border between the DG granular and molecular layers (M/G), and in the center of ii) the inner half of the DG-ML (closer to the granular layer; M-in) and iii) the outer half of the DG-ML (M-out). Finally, the PSA-NCAM immunofluorescent signal was measured in the DG hilus, DG-ML, CA4 pyramidal and CA1 stratum lacunosum-moleculare layers (Suppl. Fig. S5).

### Hippocampal BDNF level

The hippocampal BDNF levels were determined as described previously^72^, employing the *BDNF SimpleStep* commercial enzyme-linked immunosorbent assay (ELISA) kit (ab212166, Abcam) and used according to the manufacturer’s instructions. The data were normalized to the protein content measured via the use of a BCA1 kit (B9643; Sigma-Aldrich, Saint Louis, USA). The absorbance was measured using a *Tecan Infinite M200 Pro* microplate reader. The specimens were processed in duplicates and the values were averaged. The BDNF was originally measured in 8 animals per group (11–13 weeks of age; males only). Since two of the animals (1 WT and 1 SCA1) evinced distinctly lower amounts of proteins than the other mice, and two other animals (1 WT and 1 SCA1) exhibited unexplainably high differences between the duplicates (tens of %), we considered these cases to be unreliable and excluded them.

### Urinary corticosterone level measurements

Independent cohort of mice (N = 9 *WT* and 9 *SCA1*; 10 weeks ± 4 days of age; housed individually for at least 2 weeks prior to the experiment) were restrained by hand for 40 seconds (between 07:30 and 08:00) and their spontaneously released urine was sucked from a clean plastic container by means of a pipette. The procedure was repeated 65 (± 5) minutes later. The urine was stored at −80 °C. ELISA kits were used according to the manufacturer’s instructions in order to measure the corticosterone (*Arbor Assays*; K014-H5) and creatinine (*Arbor Assays*; K002-H5) levels. The absorbance was measured using a *Tecan Infinite M200 Pro* microplate reader.

### Mitochondrial high-resolution respirometry

The mitochondrial respiration was examined in the cerebellar and hippocampal tissue of the independent cohort of mice (11–13 weeks of age; N = 5 WT and 6 SCA1 mice). All the mitochondrial experiments were performed in quadruplicates (4 measurements per mouse and brain structure).

Following cervical dislocation, the whole brain was extracted rapidly and the cerebellum and one hippocampus were dissected, dried and weighed and subsequently homogenized in MiR05^73^ respiration medium using a PBI-Shredder O2k-Set (OROBOROS INSTRUMENTS, Innsbruck, Austria). The entire procedure was performed on ice and all the buffer solutions were ice-cold.

The tissue homogenates were transferred into 4 calibrated Oxygraphs (O2k, OROBOROS INSTRUMENTS, Innsbruck, Austria), each equipped with 2 chambers (2 ml). The oxygen polarography was measured at 37 °C in the O2k-chambers and the oxygen concentration (μM) as well as the oxygen flux per volume (pmol O_2_/s/ml) were recorded in real-time using DatLab software, version 7.3.0.3 (OROBOROS INSTRUMENTS, Innsbruck, Austria). The *substrate–uncoupler–inhibitor titration* (SUIT)^31^ protocol was employed (Fig. 7a, Suppl. Methods). The mitochondrial respiration was assessed in the following respiratory states: 1) complex I OXPHOS capacity in the ADP-activated state of oxidative phosphorylation (*P I*), 2) complex I + II OXPHOS capacity (*P I* + *II*), 3) maximum capacity for electron transport (*E I* + *II*), 4) complex II uncoupled capacity (*E II*) and 5) *complex IV* capacity. The mitochondrial respiration was expressed in pmol O_2_/s/mg of homogenized tissue. In subsequent analysis, the respiration was also adjusted for specific citrate synthase activity (log2 [mIU/mg]) by inclusion of the factor as a covariate into mixed-effects model (See Suppl. Methods for detailed description).

### Statistical analyses

All the statistical analyses and data visualizations were performed in *R* statistical software^74^. The parametric statistical analyses were extended by permutational or bootstrapping techniques (10 000–20 000 permutations/resamplings) to avoid parametric method assumptions. Generally, the comparisons between the SCA1 and WT mice were conducted using the permutation t-test. The effect size was determined via *Cliff*’*s d* and/or the standardized regression coefficient (*β*), and their 95% confidence intervals (CI) were based on the *bias-corrected and accelerated* (BCa) *bootstrap* method^75^ using the *effsize*^76^ and *boot*^77^ R packages. The repeated/multiple measurements data were analyzed via the permutation test of the *linear mixed-effects model* (LME), with the subject coded as a random factor (with random intercept) and with the *autoregressive 1* (AR1) variance-covariance structure in the case of the serial data, using the *nlme*^78^ and *predictmeans*^79^ R packages. The (paired) permutation t-test followed by the *False Discovery Rate* correction for multiple comparisons^80^ was used as the post-hoc test when required. The multidimensional data were analyzed via the *permutational multivariate analysis* of the variance (PERMANOVA) and visualized using *non-metric multidimensional scaling* (NMDS) using the *vegan*^81^ R package in both cases. The ROC curve, the area under the ROC curve (AUC), its 95% CI and the bootstrapping-based test of the difference between the AUCs were computed using *pROC*^82^ R package. The *linear model* (LM) or the *generalized additive model*^83^ (GAM), extended via the BCa bootstrapping of the (partial) regression coefficient(s) (*β*), were used to evaluate (dissociate) the effect(s) of the numerical predictor(s) on a single response variable. P < 0.05 was defined as significant, with emphasis on the results from resampling approaches. See Suppl. Methods for details.

## Supplementary information

Supplementary Information.

Supplementary Data1.

## Data Availability

The dataset used in this publication is available on request at f.tichanek@gmail.com
