# Supplementary material for: Hippocampal mitochondrial dysfunction and psychiatric-relevant behavioral deficits in spinocerebellar ataxia 1 mouse model
Source: Sci Rep. 2020 Mar 25;10:5418. doi: 10.1038/s41598-020-62308-0 (PMC7096488; doi:10.1038/s41598-020-62308-0)
Supplement: Supplementary file 1 — Supplementary Information. [file 41598_2020_62308_MOESM1_ESM.pdf]

Hippocampal mitochondrial dysfunction and psychiatric-relevant behavioral deficits in spinocerebellar ataxia 1 mouse model

Filip Tichanek, Martina Salomova, Jan Jedlicka, Jitka Kuncova, Pavel Pitule, Tereza Macanova, Zuzana Petrankova, Zdenek Tuma and Jan Cendelin

SUPPLEMENTARY INFORMATION

Supplementary methods

Behavioral experiments

The following behavioral tests were used for behavioral characterization:

Elevated plus maze test (EPM): Mice were placed in the center of the EPM arena (50 cm above the floor) consisting of 4 arms – 2 opposing open arms (30 x 5 cm) and 2 opposing closed arms (30 x 5 cm; 15 cm high walls). Mice were left to explore the platform for 6 minutes. We evaluated the relative time spent in the open arms, expressed in % (*EPM open arms*).

Open field (OF) test: Mice were placed in the center of a plastic opaque arena (50 x 50 x 50 cm; 3 white walls and 1 partially dark wall) to freely explore the arena for 10 minutes. We evaluated the total distance moved (*OF distance*; m), relative distance walked in < 5 cm from the arena edge from the 1st to the 10th minute (*OF thigmotaxis*; %) and relative time spent in the corners of 1 dm^2 of area, again from the 1st to the 10th minute (*OF corners*; %).

Object-location memory test (OLM): Mice were left to explore the OF arena with 4 objects that differed in color, shape and surface (~6 cm of diameter, 6 to 12 cm of height; objects labeled A, B, C, D) for 6 minutes in each session (S). The test consisted of 4 sessions, with 2 sessions in 1 day with a 5-minute-long inter-session break. During the break, the arena was cleaned with ethanol. On the 1st day (S1 and S2) and S3 of the 2nd day, the objects positions were stable. Before S4, position of 2 crosswise objects (B and D) were changed. We counted mouse entrances to objects' proximity (< 7 cm from the object; body center) and expressed relative B-D pair exploration (compared to A-C pair exploration), separately for *pre-exchange* (S1-S3) and *post-exchange* (S4) *phases* of the OLM.

Acoustic startle response (ASR): The Startle and Fear Combined System (Panlab, Spain) device was used for the whole of the experiment. The device includes a sensor that detects the strength of the movements of the mouse (whole-body as well as partial-body movement and utilizing a high-sensitivity weight transducer) in response to an acoustic stimulus (arbitrary unit; the device has been described elsewhere in details[1,2]). The mice were placed in a small plexiglass cylinder (5 x 10 x 3.5 cm) mounted on a grid floor in an acoustically-isolated box. The chamber was situated on top of a platform. Firstly, the mice were left to become habituated to the chamber for 300 seconds with a background noise of 60 dB. Next, the mice were exposed to 72 strong stimuli (120 dB, 100 ms, pseudorandom inter-stimuli intervals between 4-10 seconds). Of the 72 stimuli, 24 were preceded by a prepulse sound stimulus (80 dB, 40 ms duration; 250 ms prior to the strong stimuli) and were labeled *predictable stimuli,* whereas 48 were not preceded by any sound (*unpredictable stimuli*). We measured the maximum startle response amplitude (*startle amplitude*; arbitrary unit), ratio between the startle

response amplitudes after the *predictable* and relative to the *unpredictable* stimuli, thus reflecting the prepulse inhibition (*PPI*; %), and the average time between the *unpredictable* stimulus and the maximum startle amplitude (*startle latency*; ms). Since the mice were unable to walk during the procedure, the test was not affected by abnormal mobility of the SCA1 mice. A number of individuals from the youngest cohort (1 WT and 2 SCA1) that showed persistently frozen responses with no movement were removed from the ASR analysis.

Gait: Gait characteristics were evaluated using the DigiGait device (Mouse Specifics, Inc., MA) with continuously running belt, forcing the animals to walk at a speed set by the experimenter. We used belt speeds of 12 and 18 cm/s. For all mice and both speeds, we aimed to get 5 records with > 10 steps. Animals that were not able perform the experiment (no continuous walk with ≥ 10 steps for each of the speeds) were removed from the gait data analysis (6 weeks of age: 1 WT mouse; 10 weeks of age: 1 WT and 4 SCA1 mice; 17 weeks of age: 2 SCA1 mice; 26 weeks of age: 2 SCA1 mice). Gait parameters from multiple measurements and from the left and right legs were averaged for each individual. See Suppl. Table S1 for list of evaluated gait parameters.

Rotarod: We used the RotaRod Advanced (TSE Systems GmbH, Germany) device, with rod diameter of 3.5 cm and slow acceleration from 0 to 60 RPM within 8 minutes. Mice underwent the rotarod test on 5 consecutive days with 5 measurements per day and inter-trial interval of 20 minutes. We used day-average values of latency to fall from the rotarod (*rotarod latency*, s).

Morris water maze test (MWM): Mice learnt to locate the hidden platform (the center of the north-western quadrant) using intra-maze distant cues (pictures and symbols on arena wall) for 7 consecutive days. The mice underwent 4 trials per day, each with a different starting position and with 8-minute-long inter-trial intervals. Next, the animals underwent the probe trial (one session with starting position in the south; D8) with absent escape platform. Thereafter, mice underwent a test of navigation to the visually marked platform (two days D8 and D9) to verify that the potential learning deficit is not caused by a visual deficit or lack of motivation. The pool had a diameter of 1 meter, with water depth of 21 cm and the platform 0.5-1 cm under the water surface. Each session lasted a maximum of 1 minute. Unsuccessful mice were slowly put on to the platform and forced to stay there for 30 seconds. Water was made opaque by white non-toxic (food) coloring and was at a temperature of 26(±1)°C. We primarily measured latency to locate the platform averaged per day (*MWM latency*, s) and the proportion of time when the animal was in a non-moving state (swimming speed < 1.75 cm/s; *MWM non-moving*, %).

Water T-maze (WTM): the WTM test was performed as described elsewhere[3], with a number of modifications. The mice learned to navigate to the hidden platform (0.5 cm under the surface of the

water) placed in one of the two arms of the T-shaped arena (arm width: 7 cm; arm lengths: 38, 30 and 30 cm). The arena was filled with 15 cm of opaque water at 25(±1)°C. The test proceeded on 4 consecutive days (D) with 3 sessions (S) per day (2 on D1). Each session consisted of 10 trials. The time between the sessions was at least 1 hour. Prior to the experiment, the platform was removed and the mice were left to turn to one of the sides (repeated 5 times) so as to allow for the evaluation of the side preference. The platform was then placed on the non-preferred side for S1-S7 and reversed to the opposite side for S8-S11. After reaching the platform, the mice were left there for 15 seconds. We recorded the error rate manually (error = the mouse turned to the wrong arm first). The two sessions at the start of the experiment (S1-S2) and following the relocation of the platform (S8-S9) were considered *training* sessions, whereas the rest were considered *testing* sessions (S3-S7 and S10-S11). After turning to one of the arena arms, the mice were not allowed to return to the starting arm. If the mice demonstrated immobile behavior, they were motivated to move via noise or the gentle touching/pinching of the tail, if necessary, until the mice reached the hidden platform. We assessed the overall error rate averaged across all the *testing* sessions (*T-maze errors*, %, S3-S8 and S10-S11), the error rate in the *testing* sessions specifically during the learning phase (*T-maze learning e.*, %, S3-S8) and the error rate in the *testing* sessions following the relocation of the platform (*T-maze inflexibility*, %, S10-S11).

Forced swimming test (FST): The mice were placed in a cylindrical container (28 cm high, 18 cm diameter) filled with 15 cm of water (25±1 °C) and left there for 6 minutes. We recorded the duration of immobility across the whole of the test (*FST immobility*, %). Since the genotype-related difference in the immobility was more pronounced at the first half of the test in some age cohorts (Suppl. Fig. S1), we also evaluated immobility specifically during the 1st half of the test (*FST initial immobility*, %). Immobility was classified automatically according to a change in the pixels that reflected the area shape of the bodies of the mice (<5% pixel change averaged over 5 seconds).

The behavior in the EPM, OF, OLM, MWM and FST tests was automatically tracked and evaluated by means of EthoVision® XT 7.1 (Noldus Information Technology b.v., Netherlands). The preprocessed data obtained from the behavioral characterization are shown in Suppl. Data1, including those measured parameters that were not statistically evaluated.

Beside the basic characterization, 2 independent cohorts underwent FST (11 animals per group, aged 6 weeks) as described above or sucrose preference test (21 WT and 7 SCA1 mice, aged 11-13 weeks). The sucrose preference was performed as described elsewhere[4]. At first, separately housed mice were being habituated to 2 bottles in the cage (for 5 days). Then, one bottle was filled by 1% sucrose whereas the second contained water. Subsequently, fluids intake was measured daily for 4 days. Positions of the bottles were switched daily as well. The consumptions of both fluids were

averaged over the 4 days and sucrose preference was expressed as relative sucrose consumption over the total fluid intake (%).

Selection of the most sensitive tests of functional impairments

Out of all evaluated indicators from behavioral and motor characterization (Suppl. Table S1), we chose those which were highly sensitive towards the SCA1 genotype in at least two consecutive age cohorts. The criteria were defined by statistical significance of difference between WT and SCA1 mice ($P < 0.01$) and by Cliff's delta effect size (> 0.6 or < -0.6). If there were several strongly correlating indicators from the same test, we chose the one with the highest sensitivity and/or better interpretability.

We identified the following *sensitive indicators*: *OF distance, OF thigmotaxis*, *rotarod latency* (averaged), *MWM non-moving* (average from hidden platform phase; D1-D7); *T-maze errors* (average from S2-S7 and S10-S11) and *FST immobility*.

Histology

Firstly, we performed *preliminary analysis* in the oldest age cohort (8 animals per group). Next, the brain regions identified as genotype-sensitive were evaluated across all age cohorts (N = 8 WT and 10 SCA1 mice or 9 animals per group in case of the youngest cohort).

To assess cerebellar volume, we measured the volume of VIII, IX and *Copula pyramidalis* lobules, separately for molecular and granular layers. We used every 4th slice and grid density of 200 µm. As the caudal edge of the cerebellum was sometimes damaged (cohort aged 22 weeks at the day of death and several WT mice of the two youngest cohort), we started from the posterior part where both molecular and granular layers were present and without damage. For evaluation of hippocampal subregions' volumes, we used every 8th slice for *preliminary analysis* and every 4th slice for *follow-up analysis*. Grid density was set at 200 µm in the case of *Cornu ammonis* (CA) subregions and molecular layer of dentate gyrus (DG) volume. A grid density of 66.7 µm was used for DG polymorph and DG granular layers' volume estimation. The sampling density that we used has already been shown to be sufficient to estimate the volume of different DG subregions in mice[5]. Parietal cortex thickness was directly measured from the 2nd to the 6th cortical layers. The 1st cortical layer was ignored, as it was often damaged. The thickness was measured by 5 evenly distributed measurements per each side and slice. The measured region was defined as the cortex above the hippocampal DG where hippocampal CA extends under DG, but does not reach the lower edge of the brain. For hypoglossal nucleus volume estimation, we used every 4th slice with grid density of 200 µm. All histological data are shown in

Suppl. Data1 along with data from behavioural characterization. See Suppl. Fig. S4 for representative images.

In case of hippocampal volumes, we firstly compared absolute volumes. In the subsequent analysis, we used the brain weight as a covariate in the general linear model (supplemented by bootstrapping of partial regression coefficients) to obtain partial effect of the genotype on the hippocampal volume, adjusted for the confounding effect of the brain weight.

For evaluation of brain-behavior direct associations, we focused on the volume of the most genotype-sensitive brain regions (Cb-ML, CA-SRLM and DG-ML) and total brain weight (to investigate the association between the behavior and non-specific brain atrophy). We then evaluated the association between volumes of these brain measures and *sensitive indicators* from the behavioral characterization. Moreover, because cognitive flexibility was more impaired than initial learning during the water T-maze in young SCA1 mice and because the neurobiological substrate of the flexibility could substantially differ from the initial learning, we also explored the association of the brain measures with *T-maze inflexibility.*

Immunofluorescence

The slices were mounted in 0.01M PBS (4 x 5 minutes), incubated in blocking solution (10% normal goat serum [Abcam, ab7481], 0.3% Triton™ X-100, 90% PBS), then incubated in primary antibody solutions (primary antibodies, 5% of normal goat serum, 0.2% Triton™ X-100 and ~95% PBS). After 16 hours, the slices were repeatedly rinsed in PBS (5, 10 and 30 minutes), then incubated in secondary antibody solutions (1/400 of secondary antibodies, 5% of normal goat serum, 0.3%Triton™ X-100, ~95% PBS) for 2 hours. The slices were then rinsed in PBS (3x), incubated in DAPI for 5 minutes (1/500, Sigma-Aldrich), rinsed in PBS (3 x), collected on gelatin-subbed glass slides and coverslipped using fluorescence mounting medium (Fluoroshield™, Sigma-Aldrich).

NeuN staining was performed using mouse anti-NeuN primary antibodies (1/500, Millipore, MAB377) and Alexa Fluor® 488 anti-mouse secondary antibodies (Abcam, ab150117). With respect to the DCX and PSA-NCAM double staining, we used rabbit anti-DCX (1/4000, Abcam, ab18723) with mouse anti-PSA-NCAM (1/400, Bioscience, 14-9118-82) as the primary antibodies and Alexa Fluor® 594 goat anti-rabbit pre-adsorbed (Abcam, ab150084) and goat anti-mouse IgM cross-adsorbed (Thermofisher, A-21042) as the secondary antibodies. 2 or 4 brains were stained at once (i.e. in 1 of 3 *blocks*), with equal numbers of WT and SCA1 mice in each of the blocks. Only the brains of mice that had not previously been exposed to behavioral experiments (13-15 weeks of age; 4 males and 1 female

per group) were used for immunofluorescent staining. We used every 6th slice for each animal and staining, commencing from the 12th slice containing the hippocampus (from the frontal part); a total of 4 slices per animal. Imaging was performed using a fluorescent *Olympus BX51* microscope and an *Olympus DP70* digital camera (Olympus, Japan). Detailed images were acquired by means of an Olympus IX83 spinning disk confocal microscope (Olympus, Germany). The images were acquired using a standardized setting. The analysis was performed using *Fiji* software. The NeuN staining was analyzed by means of measuring the color intensity while subtracting the immunofluorescent intensity in neighboring sites without the presence of neuronal bodies (the CA strata oriens; Suppl. Fig. S5) and via the manual counting of the neurons in the pyramidal layers of two hippocampal sub-regions: CA1 and CA2/3. With respect to each of the assessed sub-regions, we measured the intensity in 2 to 3 pairs of evenly-distributed sampling circles with diameters of approximately half the width of the given layer in one of the hippocampi from each of the stained slices (Suppl. Fig. S5). Direct counting the density of the pyramidal neurons proceeded in the same hippocampal regions as fluorescence intensity (in at least 1 depth of focus and on at least 2500 μm^2 of the CA pyramidal layer). Since the inclusion of the *block* as a covariate improved the model fit in the case of NeuN immunofluorescence in the CA2/3 (measured via BIC; see the description of the statistical methods below), the NeuN signal in the CA2/3 hippocampal subfield was adjusted for the effect of the *block* (the factor was included in the model as a covariate).The PSA-$NCAM^{+}DCX^{+}$ neurons were counted manually in 4 of the DG subgranular layers per animal (1 DG from each of the 4 dorsal hippocampal slices stained) using image sequences containing 3 to 8 depths of focus in at least 250 µm (390 µm in average) of the DG subgranular zone per 1 evaluated hippocampus. The DCX^{+} neuronal dendrites were quantified by means of the number of crossing lines (at least 250 µm per evaluated DG; 425 µm in average) located in three positions: i) the border between the DG granular and molecular layers (*M/G*); ii) the inner half of the DG-ML (closer to the granular layer; *M-inr*); and iii) the outer half of the DG-ML (*M-out*). Finally, the PSA-NCAM immunofluorescent signal was measured in the DG hilus (DG-PL), DG-ML, CA4 pyramidal and CA1 lacunosum-moleculare (CA1-SLM) layers (Suppl. Fig. S5).

Mitochondrial respiration protocol

The substrate–uncoupler–inhibitor titration (SUIT) protocol was performed by sequential addition of the sample and the following chemicals:

1) Homogenized sample without any additions = indicator of residual oxygen consumption (*ROX*)

2) Glutamate (10 mM) and malate (5 mM) = substrates providing electrons to complex I - non-phosphorylating LEAK-respiration (state L I).

3) ADP (20 mM) = OXPHOS-capacity of CI-linked activity.

4) Cytochrome c (5 mM) = detection of mitochondrial membrane damage.

5) Pyruvate (5 mM) = substrate providing electrons to complex I (state *P I*).

6) Succinate (20 mM) = complex II substrate, OXPHOS-capacity with combined CI and II-linked substrates (state *P I+II*).

7) Stepwise titration of the carbonyl cyanide-p-trifluoromethoxyphenylhydrazone (FCCP) (1 µM) = the maximum capacity of the electron transport system (ETS; state *E I+II*).

8) Rotenone (1 µM), complex I inhibitor indicating CII-linked ETS capacity (state *E II*).

9) Antimycin A (1 µM), complex III inhibitor = background oxygen consumption from undefined sources (*ROX*).

10) Tetramethylphenylenediamine (TMPD; 5 mM), an artificial substrate for complex IV, and ascorbate (5 mM) for keeping TMPD in the reduced state.

11) Azide (50 mM) to evaluate auto-oxidation rate (ROX). The decrease in respiration after azide addition indicates *complex IV* capacity (C IV).

The oxygen concentration in the chambers was kept high enough to avoid oxygen limitation of respiration.

We evaluated respiration in steps 5-8 (states *P I*, *P I+II*, *E I+II* and *E II*; all adjusted for *ROX*) and *complex IV* capacity (the decrease of respiration between steps 10 and 11). All these parameters were adjusted for 1 mg of tissue. When respiration of one sample distinctly differed from another three samples of the same animal across at least three parameters of respiration, it was removed as outlier (a total of 4 samples for hippocampal and 2 for cerebellar data).

Measuring citrate synthase activity

The procedure is described elsewhere in detail[6]. Briefly, the assay medium consisted of 0.1 mmol/l 5,5-dithio-bis-(2-nitrobenzoic) acid, 0.25% Triton-X, 0.5 mmol/l oxalacetate, 0.31 mmol/l acetyl coenzyme A, 5 µmo/l EDTA, 5 mmol/l triethanolamine hydrochloride, and 0.1 mol/l Tris-HCl, pH 8.1. 100 µl of the mixed and homogenized chamber content were added to 900 µl of the medium. Rate of absorbance change (RAC) was measured spectrophotometrically at 412 nm and 30 °C over 200 s. The RAC was used to calculate specific citrate synthase activity in a given sample (mIU/mg of tissue). As the specific citrate synthase activity showed skewed distribution, we used log-transformed values (log [mIU/mg]).

Statistical analyses

All statistical analyses were extended by permutational or bootstrapping techniques. These approaches are less sensitive to small sample size, do not rely on assumptions of parametric methods and are more robust toward outlier values[7]. Although results from fully parametric analyses are also shown in result tables, we considered the permutational (the effect of genotype) or bootstrapping-based results (models containing a continuous predictor[s]) as more relevant.

Generally, comparisons between SCA1 and WT mice were performed by permutation t-test (20 000 Monte Carlo permutations). Data with distant outliers or highly unequal variances were compared by permutation test of differences in medians (20 000 Monte Carlo permutations). All tests were two-tailed, except for testing specific hypotheses suggested by prior experiments (FST data from experiment following basic behavioral characterization). To determine effect size of the SCA genotype, *Cliff´s d* (as non-parametric indicator of effect size), standardized regression coefficient (ß) and their 95% confidence intervals (based on bias-corrected and accelerated bootstrap) were computed, using *effsize*[8] and *boot*[9] packages in R. To obtain standardized regression coefficient (ß), we fitted the linear model with *genotype* factor (i.e. presence of SCA1; 0-1) as a predictor and the given parameter as response variable. Before the model fit, both variables were scaled (zero mean and unit variance).

For the permutation t-test, we used the following script:

```
perm.t <- function(z,y,N=20000){
yz <- c(y,z)
  len1 <- length(y);len2 <- length(z);len12 <- len1+len2
  diffs <- numeric(N+1)
  diffs[1] <- t.test(y,z)$statistic
  for(i in 2:(N+1))
  {
    idx <- sample(1:len12,size=len1,replace=F)
    yy <- yz[idx]
    zz <- yz[-idx]
    diffs[i] <-t.test(yy,zz)$statistic
  }
  sum(abs(diffs)>=abs(diffs[1]))/length(diffs)
}
```

For the permutation test for median difference we used the following script:

```
perm.median <- function(z,y,N=20000){
  yz <- c(y,z)
  len1 <- length(y)
  len2 <- length(z)
  len12 <- len1+len2
  diffs <- numeric(N+1)
  diffs[1] <- median(y,na.rm=TRUE)-median(z,na.rm=TRUE)
  for(i in 2:(N+1))
  {
    idx <- sample(1:len12,size=len1,replace=F)
    yy <- yz[idx]
    zz <- yz[-idx]
    diffs[i] <- median(yy,na.rm=TRUE)-median(zz,na.rm=TRUE)
  }
  mean(abs(diffs)>=abs(diffs[1]))
}
```

For the paired permutation t-test, we used the following script:

```
perm.pair <- function(z,y,N=20000){
  td2 <- cbind(z,y);td2 <- data.frame(td2)
  y <- td2$z-td2$y
  z<- rep(0,length(td2$y))
  yz <- c(y,z)
  len1 <- length(y);len2 <- length(z);len12 <- len1+len2
  diffs <- numeric(N+1)
  diffs[1] <- t.test(y,z)$statistic
  for(i in 2:(N+1))
  {
    idx <- sample(1:len12,size=len1,replace=F)
    yy <- yz[idx]
    zz <- yz[-idx]
    diffs[i] <-t.test(yy,zz)$statistic
  }
  sum(abs(diffs)>=abs(diffs[1]))/length(diffs)
}
```

Data of repeated/multiple measurements were analyzed by permutation test (10 000 Monte Carlo permutations) of the linear mixed-effect model (LME) with the subject coded as a random factor. For serial data with > 4 timepoints, *autoregressive 1* (AR1) variance-covariance structure was included in the model if it decreased the *Bayesian information criterion* value (measure of model parsimony; the lower BIC, the more parsimonious the model is). The analyses were performed using *nlme*[10] and *predictmeans*[11] R packages. When needed, post-hoc tests were performed by permutation t-test or paired permutation t-test (within-subject comparisons), followed by *False Discovery Rate* correction for multiple comparisons[12].

When interested in the difference of general patterns rather than the difference in a single variable (e.g. analysis of gait parameters), we used the permutational multivariate analysis of variance (PERMANOVA). All evaluated parameters were scaled to have unit variance and standardized mean before the PERMANOVA execution. The multidimensional data were visualized using *non-metric multidimensional analysis* (NMDA). Both PERMANOVA and NMDA were performed using the *vegan*[13] R package.

In order to identify those behavioral measures that might be affected by signs of ataxia in the young SCA1 mice, we examined whether the *sensitive indicators* correlated to abnormal gait. In order to reduce the multidimensionality of the gait data, we extracted the *principal components* (PCs; using the *vegan* R package) and subjected them to *principal component regression* (used for Suppl. Table S13). The PCs were based on those gait parameters that differed between the genotypes significantly in at least 1 of the young cohorts (aged ≤ 15 weeks) and the direction of the genotype-related difference was stable across both cohorts. 4 parameters were chosen: 3 describing the stride lengths and 1 representing the coefficient of variance in the stride length (hind leg, 18 cm/s). The PC1 axis correlated principally with the stride length (explaining 48% of the variance), whereas the PC2 correlated with the variance coefficient of the stride length (hind leg, 18 cm/s; explaining 27% of the variance). Only the axis with a higher effect on the given parameter is presented in Suppl. Table S13.

To visualize and compare the ability of given parameters to discriminate between WT and SCA1 mice, we visualized the ROC curve and computed the area under the ROC curve (ROC-AUC). The ROC-AUC ranges from 0.5 (i.e. the parameter does not give any clue about the genotype) to 1 (no overlap between WT and SCA1 mice). 95% CI of the ROC-AUC and the statistical difference between ROC-AUCs of given parameter vs. rotarod latency were computed using the bootstrap method. All were performed by using the *pROC*[14] R package.

To dissociate partial effects of several predictors on a single response variable, we used a *linear model* extended by bootstrapping of partial regression coefficients. If some of the covariates showed a non-linear effect, we used the *generalized additive model* (GAM), using the *mgcv* package in R. Smoothness of non-linear effect was limited by a maximum number of 3 knots. All 95% CIs for partial effects were computed by the *bias-corrected and accelerated* (BCa) bootstrap method[15] (10 000 resamplings) using the *boot*[9] package in R. The (partial) effect was considered as statistically significant if the range of 95% CI for the standardized ß coefficient did not cross zero.

The standardized respiration parameters were compared by linear mixed-effect model (LME) supplemented by a permutation test. Next, to obtain standardized ß, its 95% confidence interval and P-value based on percentile bootstrap, we scaled the fixed-effect predictor (*genotype*) and standardized respiration to have zero mean and unit variance. Next, we established the LME model using these (scaled) values. We used the *bootMer* function from the *lme4* R package[16] to obtain confidence intervals and P-values based on percentile bootstrap. See representative R code for analysis of hippocampal mitochondrial complex IV respiration:

Fitting LME model
```
c_IV_model<-lme(C_IV~genotype,random=~1|subject,data=hp)
```

Permutation test of the LME model
```
perm.model<-permmodels(c_IV_model,data=hp,nsim=10000)
```

Scaling the variables to have zero mean and unit variance
```
hp$genotype_numeric<-scale(as.numeric(hp$genotype))
hp$ c_IV_scaled<-scale(hp$c_IV)
```

New LME model with variables scaled to zero mean and unit variance to obtain standardized ß and its 95 CI
```
c_IV_model_2<-lmer(C_IV_scaled~genotype_numeric+(1|subject),data=hp)
summary(c_IV_model_2)
```

Bootstrapping of LME using *bootMer* function
```
boot_LME<-bootMer(x=c_IV_model_2,FUN=fixef,nsim=10000)
```

95% CI
```
boot.ci(boot_LME,type="perc",index=2)
```

P-value based on percentile bootstrap (the smaller of the two obtained values)
```
length(boot_LME$t[,2][boot_LME$t[,2]<0])/5000
length(boot_LME$t[,2][boot_LME$t[,2]>0])/5000
```

Supplementary Figures

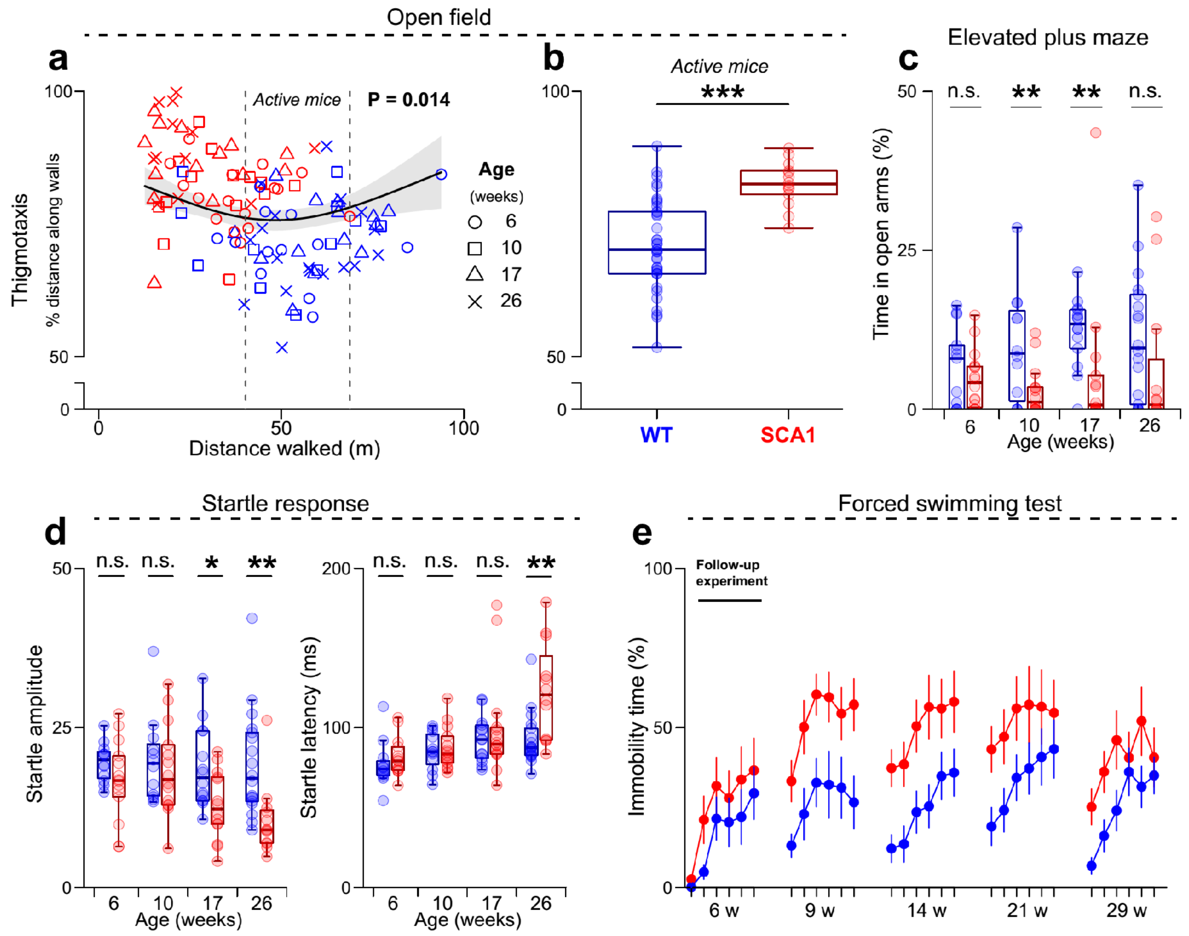

Figure S1. Psychiatric-relevant behavioral deficits in SCA1 mice.

(a) Association between thigmotaxis and locomotion during the open field test. The P-value is based on the general additive model and reflects the significance of the partial effect of the *distance moved* adjusted for *genotype*. See Methods or Suppl. Table S1 for the numbers of animals in each of the experimental groups (**a**-**d**).

(b) Thigmotaxis in 25% of the most mobile SCA1 mice and comparably mobile WT controls (distance moved*:* 40.2 – 68.71 m). N = 14 SCA1 and 38 WT mice.

(c) Time spent in the open arms during the elevated plus maze test.

(d) Maximum startle amplitude (left) and latency to maximum startle response following a startle sound not preceded by a prepulse (right).

(e) Relative time in the immobility state during the forced swimming test (FST), shown per each minute of the test (mean ± SEM). Mice aged 6 weeks were not exposed to any other behavioral tests (11 mice per group; P-values for the immobility = 0.2 [averaged over the 6 minutes] and 0.046 [averaged over 1st half of the experiment]).

Box-whisker plots (**b**-**g**) indicating the inter-quartile (IQ) intervals (box), 1.5*IQ range (whiskers) and medians (middle line). * P < 0.05, ** P < 0.01, *** P < 0.001. n.s. = not significant. Permutational t-test (**b, d-e**) or permutational test of difference in medians (**c**). Exact p-values are shown in Suppl. Table S1 (**c-e**).

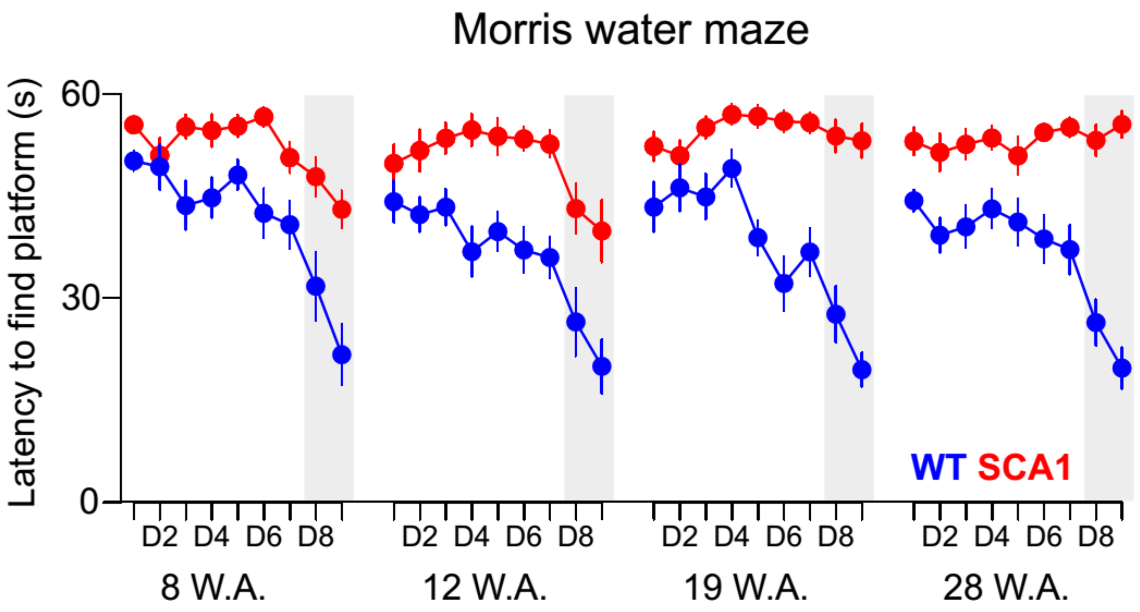

Figure S2. Altered behavior and learning during the cognitive tasks in the SCA1 mice.

Day-specific latencies to find platform during the Morris water maze test. Gray area indicates phase of the test with visually marked platform. Mean ± SEM is visualized. See Methods for the numbers of animals in each of the experimental groups.

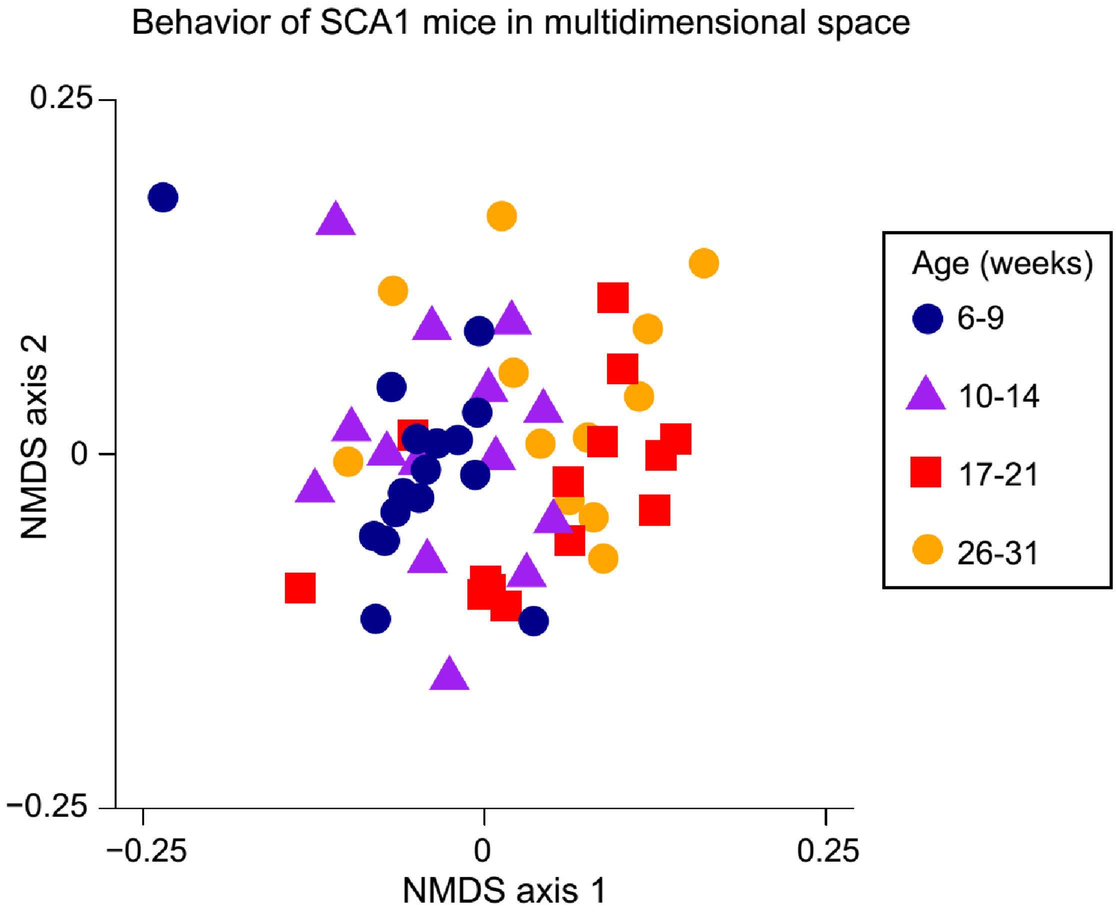

Figure S3. Non-metric multidimensional scaling (NMDS) showing the similarity of the functional impairments of SCA1 mice from differing age cohorts in multidimensional space. Each point represents 1 SCA1 mouse. The closer the points, the more similar were the mice in terms of their functional impairments (*sensitive indicators*). The significances of the effect of the age cohort on the *sensitive indicators* (based on the permutational analysis of variance, PERMANOVA) were as follows: $P < 0.001$ when all the data were used, $P = 0.65$ for the dataset that included specifically mice ≤ 14 weeks of age and $P = 0.21$ for the dataset that included specifically mice ≥ 17 weeks of age. See Suppl. Table S12 for the detailed results. See Methods for the numbers of animals in each of the experimental groups.

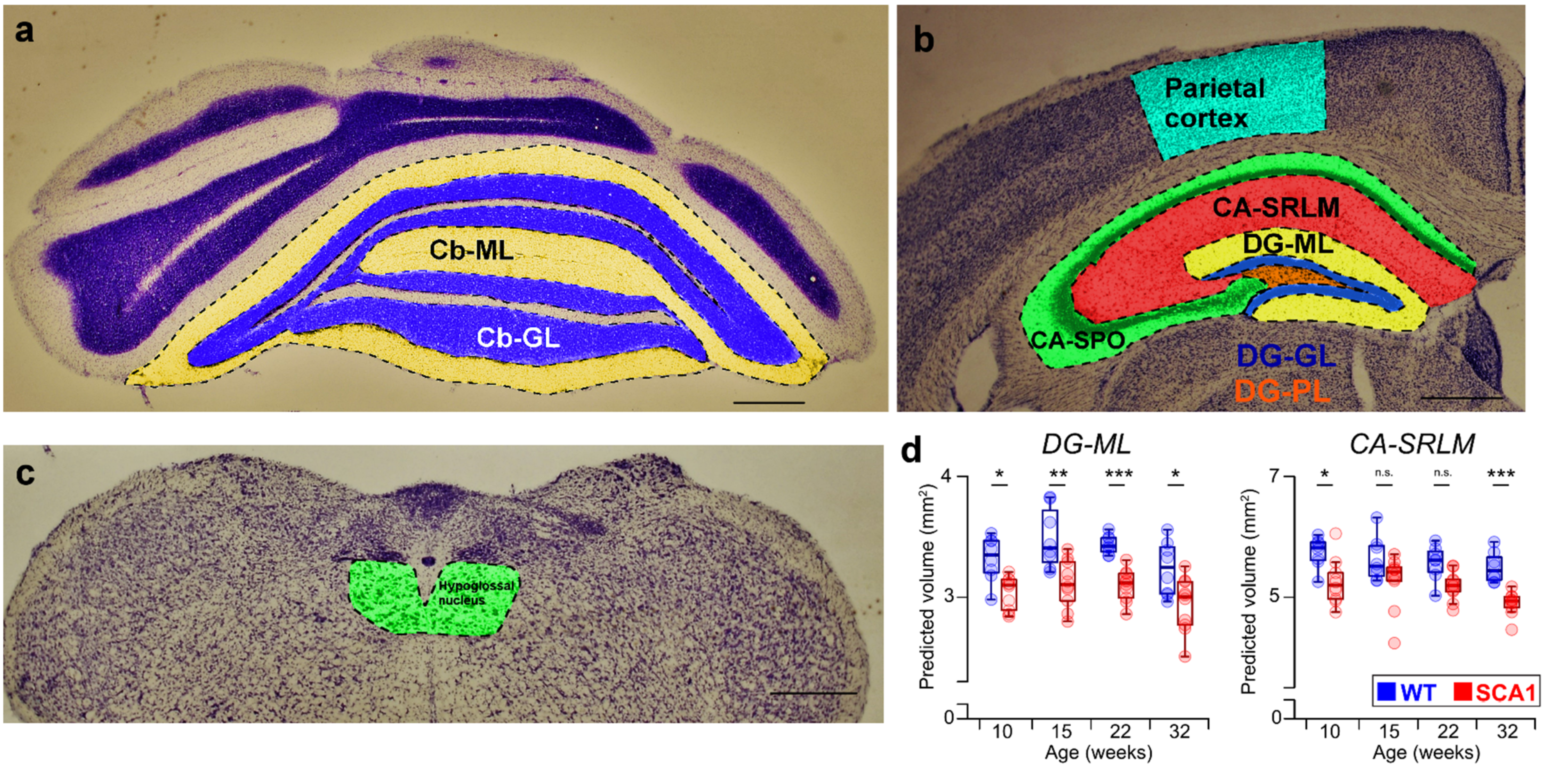

Figure S4. Representative images of the brain regions (Nissl-stained) that were histologically evaluated.

(a) Representative image of the cerebellar VIII, IX and *Copula pyramidalis* lobules including molecular (Cb-ML) and granular (Cb-GL) layers.

(b) Representative image of parietal cortex and various hippocampal subregions. CA = *Cornu ammonis.* DG = dentate gyrus. GL = granular layer. ML = molecular layer. SPO = stratum pyramidale and oriens. SRLM = stratum radiatum and lacunosum-moleculare. PL = polymorph layer (hilus).

(c) Representative image of the hypoglossal nucleus in the mouse brainstem. Scale bars (**a-c**) = 500 µm.

(d) Visualization of partial effects of genotypes on DG-ML (left) or CA-SRLM (right) volume after adjustments for the effect of total brain weight (partial residuals scaled to 400 mg), derived from general linear models (Suppl. Tables S15 and S16). The visualized values could be interpreted as expected DG-ML or CA-SRLM volume if the total brain weight of the brain was 400 mg. Box-whisker plots (**d**) indicating the inter-quartile (IQ) intervals (box), 1.5*IQ range (whiskers) and medians (middle line). Each point = 1 animal. N = 8 (WT) and 10 (SCA1) animals (9 per group in case of the youngest cohort). * $P < 0.05$, ** $P < 0.01$, *** $P < 0.001$. n.s. = not significant (permutational linear models; see Suppl. Tables S15 and S16 for detailed results).

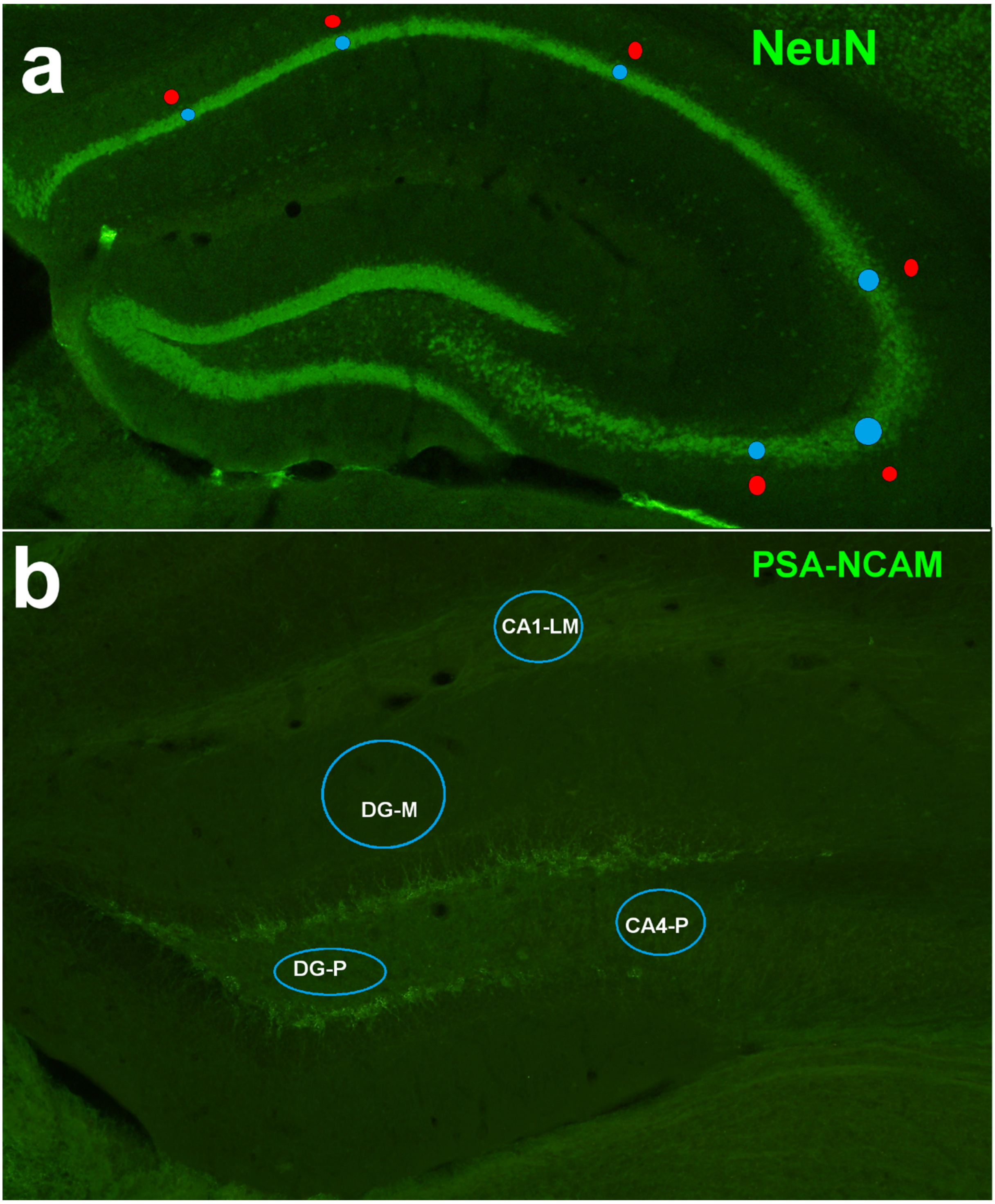

Figure S5. Impaired neurogenesis and decreased NeuN immunoreactivity in the hippocampus of the SCA1 mice

(a) Representative image of the hippocampus stained for NeuN. The circles show the area of the measurement of the fluorescence intensity (blue = measurements of interest; red = measurements of the background intensity for correction purposes) and counting of NeuN^{+} neurons.

(b) Representative image of the hippocampus stained for PSA-NCAM. The circles represent the areas where we measured the fluorescent intensity of the PSA-NCAM fluorescence signal.

Supplementary Tables

Table S1. List of indicators from characterization experiment. N = Number of successfully evaluated animals per group in given test. δ = Cliff´s non-parametric measure of effect size. ß = standardized regression coefficient for SCA1 genotype. L,U = limits of 95% confidence interval. EPM = elevated plus maze test. OF = open field test. PPI = prepulse inhibition. MWM = Morris water maze. FST = Forced swimming test. P-values are based on permutation t-test. Gait parameters were evaluated separately for hind (H) and fore (F) legs and using 2 belt speed (12 or 18 cm/s). Rotarod latency is average over all 5 days of experiment. MWM-hidden latency is average from days 2-7 of the experiment. MWM-visible latency is average from days 8 and 9. MWM non-moving is average from days 1-7. T-maze errors is average over all testing sessions (S3-S7 and S10-S11). T-maze learning e. is average error rate from all *testing* sessions of learning phase (S3-S7), whereas inflexibility reflects errors during *testing* sessions in reversal phase (S10-S11). FST initial immobility is relative time in immobility state during first 3 minutes of FST.

**a) 6-9 weeks of age**												
Behavioral parameter	**N**		**Mean**			**Cliff δ and *95% CI***			**ß and *95% CI***			
	**WT**	**SCA1**	**WT**	**SCA1**	**P**	**δ**	***L***	***U***	**ß**	***L***	***U***	
EPM open (%)	13	16	6.6	4.6	0.3282	-0.17	-0.58	0.34	-0.19	-0.58	0.18	
OF distance (m)	13	16	54.4	38.9	**0.0104**	**-0.57**	-0.83	-0.12	**-0.47**	-0.86	-0.17	
OF thigmotaxis (%)	13	16	71.5	80.4	**0.0012**	**0.66**	0.23	0.90	**0.58**	0.27	0.90	
OF adj. thigmotaxis	13	16	-0.11	-0.02	**<0.001**	**0.71**	0.28	0.92	**0.61**	0.32	0.93	
OF corners (%)	13	16	54.2	60.9	**0.0463**	0.40	-0.05	0.75	**0.36**	0.04	0.71	
PPI (%)	12	14	47.0	55.8	**0.0304**	**0.48**	0.00	0.80	**0.40**	0.09	0.80	
Startle amplitude	12	14	19.6	16.7	0.1484	0.16	-0.2	0.70	0.28	-0.07	0.65	
Startle latency (ms)	12	14	76.7	81.5	0.3744	0.38	-0.23	0.70	0.19	-0.24	0.55	
Stance/Stride (%) [H12]	12	16	77.4	76.6	0.3692	-0.29	-0.66	0.21	-0.17	-0.51	0.21	
Stride length (cm) [H12]	12	16	4.2	4.0	0.1265	-0.28	-0.65	0.21	-0.32	-0.75	0.02	
Paw angle (abs°) [H12]	12	16	20.1	21.1	0.3246	0.19	-0.30	0.60	0.19	-0.18	0.56	
Stance width (cm) [H12]	12	16	2.2	2.2	0.8556	-0.02	-0.47	0.47	0.04	-0.33	0.50	
Stride length CV [H12]	12	16	19.2	13.3	**0.0081**	**-0.57**	-0.85	-0.01	**-0.49**	-0.90	-0.19	
Stance width CV [H12]	12	16	18.6	14.1	0.3406	-0.24	-0.65	0.26	-0.20	-0.70	0.13	
Stance/Stride (%) [F12]	12	16	69.2	70.9	0.1354	0.35	-0.12	0.71	0.29	-0.08	0.65	
Stride length (cm) [F12]	12	16	4.1	3.8	0.1955	-0.25	-0.62	0.26	-0.26	-0.66	0.11	
Stride length CV [F12]	12	16	29.4	24.8	0.1794	-0.30	-0.66	0.22	-0.27	-0.66	0.13	
Stance/Stride (%) [H18]	13	16	74.0	73.1	0.5767	-0.08	-0.52	0.38	-0.11	-0.50	0.27	
Stride length (mm) [H18]	13	16	5.1	4.9	0.0902	-0.41	-0.72	0.09	-0.32	-0.66	0.04	
Paw angle (abs°) [H12]	13	16	19.2	18.8	0.6717	-0.12	-0.53	0.34	-0.08	-0.41	0.37	
Stance width (cm) [H18]	13	16	2.1	2.1	0.9718	-0.03	-0.47	0.45	0.01	-0.38	0.40	
Stride length CV [H18]	13	16	11.4	11.4	0.9995	0.19	-0.25	0.60	0.00	-0.56	0.29	
Stance width CV [H18]	13	16	16.0	18.9	0.4994	0.13	-0.33	0.54	0.13	-0.25	0.48	
Stance/Stride (%) [F18]	13	16	65.3	66.3	0.3608	0.18	-0.31	0.59	0.18	-0.20	0.55	
Stride length (cm) [F18]	13	16	5.1	4.7	**0.0177**	**-0.51**	-0.78	-0.02	**-0.45**	-0.79	-0.12	
Stride length CV [F18]	13	16	19.8	23.4	0.0829	0.30	-0.17	0.67	0.33	-0.01	0.68	
Rotarod latency (s)	13	16	178	158	0.1043	-0.41	-0.76	0.07	-0.31	-0.62	0.09	
MWM-hidden latency (s)	13	16	44.8	53.9	**0.0029**	**0.60**	0.08	0.90	**0.56**	0.21	0.86	
MWM-visible latency (s)	13	16	26.7	45.5	**0.0015**	**0.63**	0.13	0.92	**0.60**	0.22	0.87	
MWM non-moving (%)	13	16	31.3	60.3	**<0.001**	**0.65**	0.15	0.91	**0.63**	0.28	0.88	
T-maze errors (%)	13	16	10.4	30.8	**<0.001**	**0.84**	0.46	1.00	**0.74**	0.50	0.97	
T-maze learning e. (%)	13	16	4.8	18.4	**0.0024**	**0.59**	0.16	0.85	**0.45**	0.20	0.84	
T-maze inflexibility (%)	13	16	24.6	61.9	**0.0047**	**0.49**	0.00	0.82	**0.51**	0.18	0.79	
FST immobility (%)	13	16	26.4	52.5	**0.0028**	**0.61**	0.16	0.87	**0.54**	0.21	0.85	
FST initial immobility (%)	13	16	13.9	29.0	**0.0078**	**0.58**	0.14	0.84	**0.50**	0.15	0.79	

b) 10-14 weeks of age

Behavioral parameter	N		Mean			Cliff δ and *95% CI*			ß and *95% CI*		
	**WT**	**SCA1**	**WT**	**SCA1**	**P**	δ	***L***	***U***	**ß**	***L***	***U***
EPM open (%)	12	14	9.8	2.9	**0.0154**	-0.45	-0.81	0.07	**-0.48**	-0.87	-0.14
OF distance (m)	12	14	51.3	32.4	**0.0074**	**-0.58**	-0.87	-0.10	**-0.53**	-0.85	-0.17
OF thigmotaxis (%)	12	14	72.6	81.4	**0.0095**	**0.57**	0.06	0.88	**0.51**	0.15	0.84
OF adj. thigmotaxis	12	14	-0.11	-0.02	**0.0097**	**0.58**	0.11	0.88	**0.51**	0.16	0.84
OF corners (%)	12	14	60.6	63.5	0.4945	0.19	-0.32	0.62	0.14	-0.28	0.52
PPI (%)	12	14	52.6	64.8	**0.0060**	**0.58**	0.08	0.87	**0.52**	0.18	0.85
Startle amplitude	12	14	19.9	18.3	0.5792	0.17	-0.32	0.60	0.11	-0.25	0.52
Startle latency (ms)	12	14	85.0	87.1	0.6877	0.01	-0.47	0.48	0.08	-0.27	0.51
Stance/Stride (%) [H12]	11	10	79.4	79.0	0.8401	0.07	-0.57	0.48	-0.05	-0.47	0.43
Stride length (cm) [H12]	11	10	4.6	4.2	**0.0106**	**-0.65**	-0.89	-0.19	**-0.48**	-0.99	-0.19
Paw angle (abs°) [H12]	11	10	17.8	17.2	0.7277	-0.2	-0.69	0.37	-0.08	-0.45	0.42
Stance width (cm) [H12]	11	10	2.2	2.1	0.1003	-0.35	-0.76	0.22	-0.38	-0.80	0.02
Stride length CV [H12]	11	10	14.5	17.4	0.3204	0.29	-0.27	0.73	0.23	-0.20	0.66
Stance width CV [H12]	11	10	23.5	25.1	0.8213	-0.01	-0.54	0.50	0.06	-0.34	0.57
Stance/Stride (%) [F12]	11	10	70.2	67.3	0.0541	-0.4	-0.78	0.18	**-0.43**	-0.85	-0.03
Stride length (cm) [F12]	11	10	4.4	3.8	**0.0019**	**-0.73**	-0.92	-0.32	**-0.60**	-1.04	-0.31
Stride length CV [F12]	11	10	25.0	27.3	0.6043	0.05	-0.49	0.58	0.12	-0.27	0.61
Stance/Stride (%) [H18]	12	10	74.7	74.4	0.8800	-0.05	-0.56	0.49	-0.04	-0.47	0.42
Stride length (mm) [H18]	12	10	5.6	5.3	0.0596	-0.51	-0.83	0.05	**-0.41**	-0.82	-0.01
Paw angle (abs°) [H12]	12	10	17.4	16.5	0.3631	-0.15	-0.65	0.45	-0.21	-0.67	0.22
Stance width (cm) [H18]	12	10	2.0	2.1	0.4963	0.08	-0.45	0.58	0.15	-0.22	0.62
Stride length CV [H18]	12	10	10.1	14.5	**0.0449**	0.38	-0.29	0.82	**0.47**	0.04	0.85
Stance width CV [H18]	12	10	18.3	15.7	0.6063	-0.1	-0.61	0.43	-0.12	-0.50	0.38
Stance/Stride (%) [F18]	12	10	65.0	68.1	**0.0464**	0.47	-0.08	0.82	**0.45**	0.07	0.88
Stride length (cm) [F18]	12	10	5.5	5.3	0.2559	-0.24	-0.67	0.30	-0.26	-0.73	0.13
Stride length CV [F18]	12	10	22.2	24.6	0.6228	0.03	-0.50	0.58	0.12	-0.28	0.63
Rotarod latency (s)	12	14	152	124	0.1456	-0.26	-0.66	0.26	-0.31	-0.73	0.04
MWM-hidden latency (s)	12	14	39.2	53.3	**<0.001**	**0.83**	0.47	0.97	**0.75**	0.47	0.99
MWM-visible latency (s)	12	14	23.2	41.5	**0.0068**	**0.65**	0.14	0.92	**0.53**	0.16	0.84
MWM non-moving (%)	12	14	25.5	58.8	**<0.001**	**0.76**	0.36	0.95	**0.67**	0.36	0.95
T-maze errors (%)	12	14	7.7	29.9	**<0.001**	**0.88**	0.51	0.99	**0.70**	0.45	0.99
T-maze learning e. (%)	12	14	4.7	20.4	**0.0174**	**0.45**	-0.02	0.78	**0.40**	0.15	0.86
T-maze inflexibility (%)	12	14	15.4	53.6	**0.0030**	**0.50**	0.01	0.84	**0.55**	0.22	0.84
FST immobility (%)	12	14	24.2	49.5	**0.0053**	**0.61**	0.15	0.88	**0.53**	0.18	0.83
FST initial immobility (%)	12	14	8.2	21.0	**0.0042**	**0.64**	0.18	0.89	**0.55**	0.20	0.85

c) 17-21 weeks of age

Behavioral parameter	N		Mean			Cliff δ and *95% CI*			ß and *95% CI*		
	**WT**	**SCA1**	**WT**	**SCA1**	**P**	δ	***L***	***U***	**ß**	***L***	***U***
EPM open (%)	14	14	12.2	5.6	0.0525	**-0.66**	-0.89	-0.16	-0.36	-0.60	0.23
OF distance (m)	14	14	61.2	27.3	**<0.001**	**-0.93**	-0.98	-0.87	**-0.80**	-1.01	-0.55
OF thigmotaxis (%)	14	14	73.2	85.8	**<0.001**	**0.84**	0.29	1.00	**0.67**	0.35	0.92
OF adj. thigmotaxis	14	14	-0.10	0.01	**<0.001**	**0.74**	0.22	0.96	**0.61**	0.24	0.85
OF corners (%)	14	14	54.7	63.5	**0.0453**	**0.62**	0.14	0.89	0.38	-0.09	0.65
PPI (%)	14	14	47.9	59.0	0.0742	0.44	-0.07	0.79	0.34	-0.04	0.68
Startle amplitude	14	14	18.7	12.8	**0.0125**	**0.54**	0.09	0.82	**0.47**	0.15	0.81
Startle latency (ms)	14	14	92.6	100	0.4441	0.02	-0.43	0.47	0.16	-0.15	0.62
Stance/Stride (%) [H12]	14	13	77.8	80.3	0.0649	0.36	-0.14	0.72	**0.37**	0.05	0.80
Stride length (mm) [H12]	14	13	4.2	4.4	0.1591	0.3	-0.18	0.69	0.28	-0.10	0.64
Paw angle (abs°) [H12]	14	13	17.7	16.8	0.4340	-0.20	-0.61	0.29	-0.16	-0.53	0.24
Stance width (cm) [H12]	14	13	2.5	2.2	**0.0066**	**-0.61**	-0.86	-0.19	**-0.52**	-0.84	-0.20
Stride length CV [H12]	14	13	20.4	14.2	**0.0021**	**-0.60**	-0.86	-0.19	**-0.57**	-0.88	-0.26
Stance width CV [H12]	14	13	11.4	16.5	**0.0432**	**0.40**	-0.09	0.75	**0.40**	0.07	0.78
Stance/Stride (%) [F12]	14	13	66.3	66.0	0.7526	-0.12	-0.56	0.35	-0.06	-0.41	0.38
Stride length (cm) [F12]	14	13	4.1	4.2	0.3149	0.34	-0.15	0.72	0.20	-0.24	0.52
Stride length CV [F12]	14	13	28.5	29.2	0.8210	0.07	-0.43	0.54	0.05	-0.36	0.43
Stance/Stride (%) [H18]	14	12	73.6	75.5	0.4034	0.30	-0.32	0.76	0.18	-0.24	0.58
Stride length (mm) [H18]	14	12	5.4	5.6	0.4296	0.16	-0.37	0.60	0.16	-0.25	0.53
Paw angle (abs°) [H12]	14	12	16.8	15.3	0.2784	-0.27	-0.68	0.23	-0.22	-0.59	0.19
Stance width (cm) [H18]	14	12	2.5	2.2	**<0.001**	**-0.76**	-0.93	-0.39	**-0.64**	-0.93	-0.35
Stride length CV [H18]	14	12	13.0	13.0	0.9748	0.01	-0.46	0.46	0.01	-0.38	0.41
Stance width CV [H18]	14	12	10.4	12.2	0.7046	-0.04	-0.49	0.44	0.10	-0.20	0.70
Stance/Stride (%) [F18]	14	12	62.8	64.4	0.0676	0.32	-0.18	0.70	**0.34**	0.04	0.74
Stride length (cm) [F18]	14	12	5.4	5.5	0.5148	0.12	-0.37	0.57	0.13	-0.28	0.51
Stride length CV [F18]	14	12	26.8	21.9	**0.0304**	**-0.54**	-0.82	-0.05	**-0.43**	-0.76	-0.04
Rotarod latency (s)	14	14	203	92	**<0.001**	**-0.93**	-0.94	-0.88	**-0.87**	-1.06	-0.69
MWM-hidden latency (s)	14	14	41.3	55.2	**<0.001**	**0.81**	0.47	0.96	**0.70**	0.41	0.96
MWM-visible latency (s)	14	14	23.6	53.5	**<0.001**	**0.97**	0.78	1.00	**0.84**	0.62	1.03
MWM non-moving (%)	14	14	18.9	64.4	**<0.001**	**0.90**	0.60	0.99	**0.80**	0.55	1.01
T-maze errors (%)	14	14	13.6	39.8	**<0.001**	**0.90**	0.54	1.00	**0.76**	0.52	1.01
T-maze learning e. (%)	14	14	8.6	33.6	**0.0026**	**0.57**	0.05	0.87	**0.54**	0.24	0.88
T-maze inflexibility (%)	14	14	26.1	55.4	**0.0266**	0.47	-0.03	0.79	**0.43**	0.06	0.74
FST immobility (%)	14	14	31.2	51.4	0.0819	0.35	-0.13	0.71	0.34	-0.04	0.67
FST initial immobility (%)	14	14	11.8	23.6	**0.0282**	**0.48**	0.04	0.80	**0.42**	0.07	0.75

**d) 26-31 weeks of age**											
Behavioral parameter	**N**		**Mean**			**Cliff δ and *95% CI***			**ß and *95% CI***		
	**WT**	**SCA1**	**WT**	**SCA1**	**P**	**δ**	***L***	***U***	**ß**	***L***	***U***
EPM open (%)	18	12	11.2	6.3	0.2348	-0.33	-0.69	0.16	-0.23	-0.55	0.18
OF distance (m)	18	12	57.5	24.7	**<0.001**	**-0.89**	-0.98	-0.72	**-0.80**	-0.99	-0.53
OF thigmotaxis (%)	18	12	70.5	89.3	**<0.001**	**0.89**	0.59	0.99	**0.75**	0.52	0.99
OF adj. thigmotaxis	18	12	-0.12	0.04	**<0.001**	**0.84**	0.54	0.97	**0.71**	0.44	0.94
OF corners (%)	18	12	53.3	71.0	**<0.001**	**0.71**	0.20	0.94	**0.64**	0.32	0.95
PPI (%)	18	12	42.7	76.4	**<0.001**	**0.79**	0.38	0.95	**0.67**	0.39	0.92
Startle amplitude	18	12	19.2	10.5	**0.0027**	**0.73**	0.26	0.92	**0.51**	0.23	0.8
Startle latency (ms)	18	12	92.4	123	**0.0042**	**0.68**	0.25	0.90	**0.56**	0.23	0.92
Stance/Stride (%) [H12]	18	12	79.5	83.1	0.0507	0.42	-0.09	0.78	0.38	0.00	0.71
Stride length (mm) [H12]	18	12	4.6	4.3	**0.0302**	-0.45	-0.76	0.04	**-0.40**	-0.73	-0.06
Paw angle (abs°) [H12]	18	12	20.5	13.7	**<0.001**	**-0.88**	-0.97	-0.64	**-0.69**	-0.94	-0.46
Stance width (cm) [H12]	18	12	2.6	2.4	0.1139	-0.31	-0.67	0.11	-0.27	-0.60	0.03
Stride length CV [H12]	18	12	14.1	13.3	0.7007	-0.10	-0.52	0.35	-0.07	-0.43	0.29
Stance width CV [H12]	18	12	10.7	11.3	0.8320	-0.14	-0.59	0.36	0.04	-0.30	0.51
Stance/Stride (%) [F12]	18	12	65.2	65.7	0.7880	0.13	-0.38	0.58	0.06	-0.40	0.42
Stride length (cm) [F12]	18	12	4.5	4.2	0.1535	-0.31	-0.68	0.17	-0.27	-0.61	0.10
Stride length CV [F12]	18	12	31.1	27.0	0.2722	-0.27	-0.69	0.24	-0.23	-0.64	0.15
Stance/Stride (%) [H18]	18	10	75.7	80.4	**0.0051**	**0.59**	0.14	0.85	**0.51**	0.17	0.81
Stride length (mm) [H18]	18	10	5.9	5.4	**<0.001**	**-0.73**	-0.93	-0.43	**-0.61**	-0.97	-0.32
Paw angle (abs°) [H12]	18	10	19.3	15.1	**0.0031**	**-0.61**	-0.86	-0.20	**-0.51**	-0.78	-0.22
Stance width (cm) [H18]	18	10	2.5	2.3	**0.0475**	**-0.44**	-0.77	-0.01	**-0.36**	-0.67	-0.02
Stride length CV [H18]	18	10	10.7	12.3	0.4419	0.23	-0.27	0.64	0.19	-0.13	0.82
Stance width CV [H18]	18	10	8.9	9.9	0.6329	0.23	-0.25	0.63	0.10	-0.30	0.42
Stance/Stride (%) [F18]	18	10	64.0	64.9	0.4668	0.23	-0.36	0.71	0.17	-0.33	0.58
Stride length (cm) [F18]	18	10	5.9	5.4	**0.0314**	-0.44	-0.79	0.03	**-0.47**	-0.88	-0.09
Stride length CV [F18]	18	10	25.2	29.4	0.1510	0.36	-0.16	0.74	0.28	-0.13	0.60
Rotarod latency (s)	18	12	205	92.6	**<0.001**	**-0.90**	-0.95	-0.79	**-0.81**	-1.00	-0.62
MWM-hidden latency (s)	18	12	40.0	53.0	**<0.001**	**0.86**	0.46	0.98	**0.65**	0.43	0.91
MWM-visible latency (s)	18	12	23.1	54.4	**<0.001**	**0.90**	0.48	1.00	**0.82**	0.59	0.97
MWM non-moving (%)	18	12	27.9	46.3	**0.0161**	**0.53**	0.08	0.81	**0.44**	0.10	0.75
T-maze errors (%)	18	12	11.2	34.8	**<0.001**	**0.89**	0.60	0.98	**0.76**	0.53	1.04
T-maze learning e. (%)	18	12	8.8	31.0	**<0.001**	**0.72**	0.30	0.93	**0.63**	0.33	0.97
T-maze inflexibility (%)	18	12	17.2	44.2	**0.0054**	**0.51**	-0.03	0.86	**0.55**	0.17	0.85
FST immobility (%)	18	12	23.0	38.0	0.0897	0.36	-0.14	0.74	0.34	-0.03	0.71
FST initial immobility (%)	18	12	7.0	16.5	**0.0189**	**0.54**	0.07	0.83	**0.46**	0.10	0.80

Table S2. Permutational linear mixed-effect models describing relative exploration of objects pairs during object-location memory test in relation to *phase* (before the change of objects' position [*pre-exchange*] or after the change [*post-exchange*]), *genotype* (WT or SCA1) and their interaction. Individual subjects are included as a random factor.

W.A. = weeks of age. *d.f.* = degrees of freedom. *d.d.f.* = denominator degrees of freedom. P = significance derived from *linear mixed-effect model* (LME). Perm. P = significance from permutation test of LME model (10 000 free permutations).

(a) 6 W.A.	Variable	*d.f.*	*d.d.f.*	F	P	Perm. P
	intercept	1	27	1364		
	Phase	1	27	6.7	0.0156	0.0134
	Genotype	1	27	1.1	0.2959	0.2969
	*Phase*Genotype*	1	27	3.3	0.0808	0.0722
(b) 10 W.A.	Variable	*d.f.*	*d.d.f.*	F	P	Perm. P
	intercept	1	24	854		
	Phase	1	24	23.5	<0.001	<0.001
	Genotype	1	24	1.9	0.1801	0.1642
	*Phase*Genotype*	1	24	3.4	0.0767	0.0686
(c) 17 W.A.	Variable	*d.f.*	*d.d.f.*	F	P	Perm. P
	intercept	1	26	757		
	Phase	1	26	0.6	0.4339	0.4342
	Genotype	1	26	0.06	0.805	0.8101
	*Phase*Genotype*	1	26	0.19	0.6671	0.6738
(d) 26 W.A.	Variable	*d.f.*	*d.d.f.*	F	P	Perm. P
	intercept	1	28	1078		
	Phase	1	28	13.5	0.001	<0.001
	Genotype	1	28	1.8	0.1858	0.1944
	*Phase*Genotype*	1	28	0.05	0.8195	0.8261

Table S3. Permutational linear mixed-effect models with autoregressive 1 variance-covariance structure modeling latency to reach platform during Morris water maze test. Individual subjects represent a random factor.

W.A. = weeks of age. *d.f.* = degrees of freedom. *d.d.f.* = denominator degrees of freedom. P = significance derived from LME. Perm. P = significance from permutation test of LME model.

**(a)** 8 W.A.	Variable	*d.f.*	*d.d.f.*	F	P	Perm. P
	intercept	1	216	1375.9521		
	Day	8	216	10.2172	<0.001	<0.001
	Genotype	1	27	12.201	0.0017	0.002
	*Day*Genotype*	8	216	3.183	0.002	0.0044
**(b)** 12 W.A.	Variable	*d.f.*	*d.d.f.*	F	P	Perm. P
	intercept	1	192	787.7302		
	Day	8	192	10.0637	<0.001	<0.001
	Genotype	1	24	20.2114	<0.001	<0.001
	*Day*Genotype*	8	192	1.4877	0.1639	0.1608
**(c)** 19 W.A.	Variable	*d.f.*	*d.d.f.*	F	P	Perm. P
	intercept	1	208	1274.7291		
	Day	8	208	7.4903	<0.001	<0.001
	Genotype	1	26	46.3835	<0.001	<0.001
	*Day*Genotype*	8	208	6.527	<0.001	<0.001
**(d)** 28 W.A.	Variable	*d.f.*	*d.d.f.*	F	P	Perm. P
	intercept	1	224	1201.2753		
	Day	8	224	5.703	<0.001	<0.001
	Genotype	1	28	45.4089	<0.001	<0.001
	*Day*Genotype*	8	224	4.9505	<0.001	<0.001

Table S4. Table of contrasts (post-hoc tests) showing day-specific significance of difference in latency to reach platform during Morris water maze test between genotypes (WT vs. SCA1; first 9 rows) and within-subject comparisons between given days (D) of the experiment. The statistical significance is based on permutation t-test or paired permutation t-test (within-subject comparison). Tests were followed by *false discovery rate* correction for multiple comparison (Adj. P). W.A. = weeks of age.

	**8 W.A.**		**12 W.A.**		**19 W.A.**		**28 W.A.**	
Contrast	Raw P	Adj. P	Raw P	Adj. P	Raw P	Adj. P	Raw P	Adj. P
D1	0.0136	**0.0268**	0.0707	0.0836	0.0481	0.0695	0.0022	**0.0048**
D2	0.6897	0.6897	0.0138	**0.0180**	0.2648	0.2648	0.0039	**0.0063**
D3	0.0065	**0.0210**	0.0025	**0.0047**	0.0092	**0.0160**	0.0047	**0.0067**
D4	0.0145	**0.0268**	<0.001	**0.0010**	0.0099	**0.0160**	0.0039	**0.0063**
D5	0.0165	**0.0268**	0.0010	**0.0026**	<0.001	**<0.001**	0.0390	0.0506
D6	<0.001	**0.0039**	<0.001	**<0.001**	<0.001	**<0.001**	<0.001	**<0.001**
D7	0.0242	**0.0349**	0.0001	**0.0009**	<0.001	**<0.001**	<0.001	**<0.001**
D8	0.0120	**0.0268**	0.0074	**0.0107**	<0.001	**<0.001**	<0.001	**<0.001**
D9	<0.001	**0.0039**	0.0020	**0.0043**	<0.001	**<0.001**	<0.001	**<0.001**
D1:D7 SCA1	0.0274	**0.0356**	0.4767	0.4767	0.1725	0.2039	0.2889	0.3130
D1:D7 WT	0.0470	0.0509	0.0813	0.0881	0.2027	0.2196	0.0634	0.0750
D7:D9 SCA1	0.0392	**0.0464**	<0.001	**0.0026**	0.1298	0.1687	0.8157	0.8157
D7:D9 WT	<0.001	**0.0012**	0.0052	**0.0084**	0.0000	**0.0002**	<0.001	**<0.001**

Table S5. Permutational linear mixed-effect models with autoregressive 1 variance-covariance structure modeling relative non-moving time during Morris water maze test. Individual subjects represent a random factor.

W.A. = weeks of age. *d.f.* = degrees of freedom. *d.d.f.* = denominator degrees of freedom. P = significance derived from LME. Perm. P = significance from permutation test of LME model.

**(a)** 8 W.A.	Variable	*d.f.*	*d.d.f.*	F	P	Perm. P
	intercept	1	216	1375.9521		
	Day	8	216	10.2172	<0.001	<0.001
	Genotype	1	27	12.201	0.0017	0.002
	*Day*Genotype*	8	216	3.183	0.002	0.0044
**(b)** 12 W.A.	Variable	*d.f.*	*d.d.f.*	F	P	Perm. P
	intercept	1	192	787.7302		
	Day	8	192	10.0637	<0.001	<0.001
	Genotype	1	24	20.2114	<0.001	<0.001
	*Day*Genotype*	8	192	1.4877	0.1639	0.1608
**(c)** 19 W.A.	Variable	*d.f.*	*d.d.f.*	F	P	Perm. P
	intercept	1	208	1274.7291		
	Day	8	208	7.4903	<0.001	<0.001
	Genotype	1	26	46.3835	<0.001	<0.001
	*Day*Genotype*	8	208	6.527	<0.001	<0.001
**(d)** 28 W.A.	Variable	*d.f.*	*d.d.f.*	F	P	Perm. P
	intercept	1	224	1201.2753		
	Day	8	224	5.703	<0.001	<0.001
	Genotype	1	28	45.4089	<0.001	<0.001
	*Day*Genotype*	8	224	4.9505	<0.001	<0.001

Table S6. Table of contrasts (post-hoc tests) showing day-specific significance of difference in relative non-moving time during Morris water maze test between genotypes (WT vs. SCA1; first 9 rows) and within-subject comparisons between given days (D) of the experiment. The statistical significance is based on permutation t-test or paired permutation t-test (within-subject comparison). Tests were followed by *false discovery rate* correction for multiple comparison (Adj. P). W.A. = weeks of age.

	**8 W.A.**		**12 W.A.**		**19 W.A.**		**28 W.A.**	
Contrast	Raw P	Adj. P	Raw P	Adj. P	Raw P	Adj. P	Raw P	Adj. P
D1	<0.001	**0.0018**	0.0011	**0.0015**	0.0014	**0.0023**	0.0036	**0.0093**
D2	<0.001	**0.0018**	<0.001	**<0.001**	0.0317	**0.0457**	0.0026	**0.0084**
D3	<0.001	**0.0018**	<0.001	**<0.001**	0.0420	0.0546	0.0011	**0.0047**
D4	<0.001	**0.0028**	<0.001	**<0.001**	0.0012	**0.0023**	0.0122	**0.0177**
D5	<0.001	**0.0018**	<0.001	**<0.001**	<0.001	**<0.001**	0.0053	**0.0115**
D6	0.0046	**0.0066**	<0.001	**<0.001**	<0.001	**<0.001**	0.0364	**0.0430**
D7	0.0145	**0.0172**	<0.001	**<0.001**	<0.001	**<0.001**	0.0078	**0.0144**
D8	<0.001	**0.0018**	<0.001	**<0.001**	<0.001	**<0.001**	0.0108	**0.0175**
D9	0.0013	**0.0024**	<0.001	**<0.001**	<0.001	**<0.001**	0.0348	**0.0430**
D1:D7 SCA1	0.0676	0.0733	<0.001	**<0.001**	<0.001	**<0.001**	<0.001	**<0.001**
D1:D7 WT	0.0098	**0.0127**	0.3516	0.3516	0.7192	0.7192	<0.001	**<0.001**
D7:D9 SCA1	<0.001	**0.0018**	0.0261	**0.0309**	0.4687	0.5077	0.4992	0.4992
D7:D9 WT	0.7775	0.7775	0.0498	0.0539	0.4393	0.5077	0.0931	0.1008

Table S7. Permutational linear mixed-effect models with autoregressive 1 variance-covariance structure modeling error rate during the water T-maze test.

W.A. = weeks of age. *d.f.* = degrees of freedom. *d.d.f.* = denominator degrees of freedom. P = significance derived from LME. Perm. P = significance from permutation test of LME model.

**(a)** 9 W.A.	Variable	*d.f.*	*d.d.f.*	F	P	Perm. P
	intercept	1	270	425.4		
	Session	10	270	41.6	<0.001	<0.001
	Genotype	1	27	11.4	0.0022	<0.001
	*Session*Genotype*	10	270	2.7	0.0034	0.0016
**(b)** 13 W.A.	Variable	*d.f.*	*d.d.f.*	F	P	Perm. P
	intercept	1	240	249.3222		
	Session	10	240	35.5306	<0.001	0.005
	Genotype	1	24	9.269	0.0056	0.01
	*Session*Genotype*	10	240	2.4731	0.0078	0.005
**(c)** 20 W.A.	Variable	*d.f.*	*d.d.f.*	F	P	Perm. P
	intercept	1	260	326.6989		
	Session	10	260	16.9003	<0.001	<0.001
	Genotype	1	26	11.1879	0.0025	0.001
	*Session*Genotype*	10	260	3.3454	<0.001	<0.001
**(d)** 29 W.A.	Variable	*d.f.*	*d.d.f.*	F	P	Perm. P
	intercept	1	280	447.5031		
	Session	10	280	21.8456	<0.001	<0.001
	Genotype	1	28	21.3227	<0.001	<0.001
	*Session*Genotype*	10	280	7.0813	<0.001	<0.001

Table S8. Table of contrasts (post-hoc tests) showing significance of difference in error rate during the water T-maze test between WT and SCA1 mice, specifically for each session (S) of the experiment. The statistical significances are based on permutation t-test. Tests were followed by *false discovery rate* correction for multiple comparison (Adj. P). W.A. = weeks of age.

	**9 W.A.**		**13 W.A.**		**20 W.A.**		**29 W.A.**	
Contrast	Raw P	Adj. P	Raw P	Adj. P	Raw P	Adj. P	Raw P	Adj. P
S1	0.3582	0.3940	0.7957	0.7957	0.5319	0.5851	0.6599	0.6738
S2	0.0027	0.0293	0.0637	0.1709	0.1193	0.1641	0.0079	**0.0173**
S3	0.0223	0.0529	0.0131	0.0723	0.0066	**0.0243**	<0.001	**0.0021**
S4	0.0087	**0.0431**	0.1243	0.1919	0.0060	**0.0243**	0.0186	**0.0341**
S5	0.2278	0.2784	0.0514	0.1709	0.0417	0.0765	0.0849	0.1167
S6	0.0627	0.1150	0.1324	0.1919	0.0291	0.0641	<0.001	**0.0010**
S7	0.0240	0.0529	0.1396	0.1919	0.0033	**0.0243**	0.0016	**0.0058**
S8	0.1126	0.1548	0.4902	0.5991	0.2138	0.2614	0.1055	0.1290
S9	0.7592	0.7592	0.6950	0.7645	0.7759	0.7759	0.6738	0.6738
S10	0.1057	0.1548	0.0777	0.1709	0.0948	0.1489	0.0789	0.1167
S11	0.0118	**0.0431**	0.0018	**0.0199**	0.0195	0.0536	0.0030	**0.0081**

Table S9. Permutational linear mixed-effect models with autoregressive 1 variance-covariance structure modeling latency on accelerating rotarod.

W.A. = weeks of age. *d.f.* = degrees of freedom. *d.d.f.* = denominator degrees of freedom. P = significance derived from LME. Perm. P = significance from permutation test of LME model.

**(a)** 7 W.A.	Variable	*d.f.*	*d.d.f.*	F	P	Perm. P
	intercept	1	108	613.0018		
	Day	4	108	8.9994	<0.001	<0.001
	Genotype	1	27	1.2183	0.2794	0.2486
	*Day*Genotype*	4	108	2.9436	0.0236	0.0158
**(b)** 11 W.A.	Variable	*d.f.*	*d.d.f.*	F	P	Perm. P
	intercept	1	96	903.682		
	Day	4	96	15.7475	<0.001	<0.001
	Genotype	1	24	1.947	0.1757	0.1536
	*Day*Genotype*	4	96	0.064	0.9923	0.9908
**(c)** 18 W.A.	Variable	*d.f.*	*d.d.f.*	F	P	Perm. P
	intercept	1	104	2363.8692		
	Day	4	104	18.2746	<0.001	<0.001
	Genotype	1	26	88.5653	<0.001	<0.001
	*Day*Genotype*	4	104	4.7874	0.0014	<0.001
**(d)** 27 W.A.	Variable	*d.f.*	*d.d.f.*	F	P	Perm. P
	intercept	1	112	1844.6508		
	Day	4	112	20.838	<0.001	<0.001
	Genotype	1	28	59.3181	<0.001	<0.001
	*Day*Genotype*	4	112	3.9682	0.0048	0.0016

Table S10. Table of contrasts (post-hoc tests) showing day-specific significance of difference in rotarod latency between genotypes (WT vs. SCA1; first 5 rows) and within-subject comparisons between the first and the last day (D) of experiment. Contrast were computed by permutation t-test or paired permutation t-test (within-subject comparison). Tests were followed by *false discovery rate* correction for multiple comparison (Adj. P). W.A. = weeks of age.

	**7 W.A.**		**11 W.A.**		**18 W.A.**		**27 W.A.**	
Contrast	Raw P	Adj. P	Raw P	Adj. P	Raw P	Adj. P	Raw P	Adj. P
D1	0.8492	0.8512	0.2398	0.2822	<0.001	**<0.001**	<0.001	**<0.001**
D2	0.0287	0.1042	0.1500	0.2822	<0.001	**<0.001**	<0.001	**<0.001**
D3	0.0627	0.1265	0.1524	0.2822	<0.001	**<0.001**	<0.001	**<0.001**
D4	0.0819	0.1265	0.1818	0.2822	<0.001	**<0.001**	<0.001	**<0.001**
D5	0.5664	0.6578	0.2337	0.2998	<0.001	**<0.001**	<0.001	**<0.001**
D1:D5 WT	0.0281	0.1042	0.0042	**0.0147**	<0.001	**<0.001**	<0.001	**<0.001**
D1:D5 SCA1	0.0869	0.1265	0.0013	**0.0093**	0.0013	**0.0013**	0.0026	**0.0026**

Table S11. Results of permutational multivariate analysis of variance (PERMANOVA) describing similarity in general gait pattern (WT vs. SCA1 mice) on the basis of gait parameters measured using DigiGait device.

W.A. = weeks of age. *d.f.* = degrees of freedom. *s.s.* = sum of squares. *m.s.* = mean squares.

**(a)** 6 W.A.	Variable	*d.f.*	*s.s.*	*m.s.*	F	R^2	P
	Genotype	1	0.01	0.01	1.56	0.06	0.1068
	Residuals	26	0.17	0.0064		0.94	
**(b)** 10 W.A.	Variable		*s.s.*	*m.s.*	F		P
	Genotype	1	0.01	0.01	1.74	0.08	0.0726
	Residuals	19	0.12	0.0064		0.92	
**(c)** 17 W.A.	Variable		*s.s.*	*m.s.*	F		P
	Genotype	1	0.02	0.02	3.88	0.14	<0.001
	Residuals	24	0.14	0.0059		0.86	
**(d)** 26 W.A.	Variable		*s.s.*	*m.s.*	F		P
	Genotype	1	0.03	0.03	5.04	0.16	<0.001
	Residuals	26	0.15	0.0059		0.84	

Table S12. Results of permutational multivariate analysis of variance (PERMANOVA) comparing pattern of functional impairments in SCA1 mice of different age cohorts. (**a**) All age cohorts together. (**b**) *Pre-ataxic* mice only (age ≤ 14 weeks of age). (**c**) *Ataxic* mice only (age ≥ 17 weeks of age).

W.A. = weeks of age. *d.f.* = degrees of freedom. *s.s.* = sum of squares. *m.s.* = mean squares.

**(a)** all SCA1	Variable	*d.f.*	*s.s.*	*m.s.*	F	R^2	P
	Age	3	0.068	0.023	3.6	0.17	<0.001
	Residuals	52	0.329	0.006		0.83	
**(b)** ≤ 14W.A.	Variable		*s.s.*	*m.s.*	F		P
	Age	1	0.005	0.005	0.7	0.02	0.65
	Residuals	28	0.209	0.008		0.98	
**(c)** ≥ 17 W.A.	Variable		*s.s.*	*m.s.*	F		P
	Age	1	0.010	0.010	1.5	0.06	0.21
	Residuals	24	0.162	0.007		0.94≤	

Table S13. Linear models describing an effect of indicators potentially sensitive to motor deficits (rotarod latency and gait) or propensity to inactivity (distance moved in the OF and MWM non-moving) on the *sensitive indicators* in young (≤ 14 weeks of age) SCA1 mice. In case of gait, we extracted the principal components (PCs) and subjected them to *principal component regression*. The PCs were based on those gait parameters that differed between the genotypes significantly in at least 1 of the young cohorts and the direction of the genotype-related difference was stable across both cohorts. 4 parameters were chosen: 3 describing the stride lengths and 1 representing the coefficient of variance in the stride length (hind leg, 18 cm/s). The PC1 axis correlated principally with the stride length (explaining 48% of the variance), whereas the PC2 correlated with the variance coefficient of the stride length (hind leg, 18 cm/s; explaining 27% of the variance). Only the PC with a higher effect on the given parameter is presented. See Suppl. Methods for details.

d.f. = degrees of freedom. *ß* = standardized regression coefficient. CI = limits for 95% confidence intervals (CI-L: lower limit, CI-U: upper limit) based on bias-corrected and accelerated (BCa) bootstrap. P = significance based on parametric approach. boot. P = significance based on percentile bootstrap.

Model	$F_{d.f.}$	*ß*	CI-L	CI-U	P	boot. P
OF distance ~ Rotarod latency	$4.1_{1,28}$	0.36	-0.06	0.72	0.052	0.20
OF thigmotaxis ~ Rotarod latency	$0.00_{1,28}$	0.01	-0.29	0.64	0.98	0.97
MWM non-moving ~ Rotarod latency	$2.6_{1,28}$	-0.29	-0.77	0.24	0.12	0.41
T-maze errors rate ~ Rotarod latency	$0.26_{1,28}$	-0.1	-0.37	0.27	0.61	0.47
FST immobility ~ Rotarod latency	$1_{1,28}$	-0.09	-0.46	0.40	0.63	0.71
**Rotarod latency ~ Gait PC2**	$11_{1,24}$	**-0.56**	**-0.98**	**-0.25**	0.003	**0.003**
**OF distance ~ Gait PC2**	$4.9_{1,24}$	**-0.4**	**-0.81**	**-0.04**	0.045	0.062
OF thigmotaxis ~ Gait PC2	$0.02_{1,24}$	0.03	-0.39	0.47	0.9	0.86
MWM non-moving ~ Gait PC2	$2.2_{1,24}$	0.29	-0.1	0.8	0.15	0.22
T-maze errors rate ~ Gait PC1	$2_{1,24}$	-0.28	-0.67	0.22	0.17	0.19
FST immobility ~ Gait PC2	$0.7_{1,24}$	0.24	-0.26	0.52	0.42	0.39
OF thigmotaxis ~ OF distance	$0.3_{1,28}$	-0.1	-0.39	0.24	0.58	0.46
**MWM non-moving ~ OF distance**	$12.6_{1,28}$	**-0.56**	**-0.93**	**-0.19**	0.001	**0.004**
T-maze errors rate ~ OF distance	$0.93_{1,28}$	0.17	-0.18	0.70	0.35	0.42
FST immobility ~ OF distance	$1.25_{1,28}$	-0.2	-0.57	0.20	0.27	0.33
T-maze errors rate ~ MWM non-mov.	$0.07_{1,28}$	0.05	-0.41	0.37	0.80	0.78

Table S14. Brain regions that were evaluated in terms of their volume or thickness in WT and SCA1 mice (32 weeks of age; N = 8 animals/group; see Suppl. Fig. S4 for representative images).

WT, SCA1 = genotype-specific means (mm^3 or mm). ß = standardized regression coefficient for SCA1 genotype. CI = limits for 95% confidence intervals (CI-L: lower limit, CI-U: upper limit) based on BCa bootstrap. perm.P = significance based on permutational t-test. CA = *Cornu ammonis*. DG = dentate gyrus.

Brain area	WT	SCA1	*ß*	CI-L	CI-U	perm. P
Cerebellar granular layer	2.5	2.4	-0.24	-0.81	0.20	0.3691
**Cerebellar molecular layer**	2.9	2.1	**-0.77**	**-1.14**	**-0.50**	**<0.001**
**Molecular/granular cb. layers**	1.16	0.87	**-0.92**	**-1.10**	**-0.74**	**<0.001**
Hypoglossal nucleus	0.0144	0.0150	0.18	-0.27	0.75	0.4913
Parietal cortex thickness	0.534	0.524	-0.11	-0.61	0.40	0.6691
CA strata pyr. + oriens (SPO)	4.32	4.04	-0.27	-0.79	0.18	0.3027
**CA strata rad. + lac. mol.**	5.1	4.4	-0.85	**-1.09**	**-0.58**	**<0.001**
DG granular layer	0.61	0.57	-0.28	-0.85	0.27	0.7433
DG polymorph layer	0.56	0.55	-0.12	-0.62	0.42	0.4710
**DG molecular layer**	3.0	2.6	**-0.69**	**-1.08**	**-0.34**	**0.0043**

Table S15. Results of general linear models (LMs) describing effect of total brain weight and SCA1 genotype on the volume of stratum radiatum and lacunosum-moleculare of *Cornu ammonis* (CA-SRLM). (**a-d**) Age cohort-specific models (N = 8 WT and 10 SCA1 mice). (**e-f**) Models are based on merged data from two age cohorts (N = 17 WT and 19 SCA1 mice).

ß = standardized regression coefficient. CI = limits for 95% confidence intervals (CI-L: lower limit, CI-U: upper limit) based on BCa bootstrap. P = significance based on parametric approach. boot. P = significance based on percentile bootstrap.

(a) 10 W.A.	*ß*	CI-L	CI-U	P	boot. P
Brain weight	0.34	-0.1	0.76	0.12	0.13
Genotype	-0.5	-0.93	0.01	0.036	**0.028**
(b) 15 W.A.	*ß*	CI-L	CI-U	P	boot. P
Brain weight	0.55	0.12	0.91	0.022	0.01
Genotype	**-0.33**	**-0.84**	**-0.04**	0.12	**0.041**
(c) 22 W.A.	*ß*	CI-L	CI-U	P	boot. P
Brain weight	0.44	0.05	0.09	0.073	0.055
Genotype	-0.44	-0.83	0.09	0.075	0.068
(d) 32 W.A.	*ß*	CI-L	CI-U	P	boot. P
Brain weight	0.3	-0.09	0.5	0.052	0.076
Genotype	**-0.72**	**-1.08**	**-0.48**	<0.001	**<0.001**
(e) ≤ 15 W.A.	*ß*	CI-L	CI-U	P	boot. P
Brain weight	0.48	0.26	0.7	0.002	<0.001
Genotype	**-0.38**	**-0.69**	**-0.15**	0.009	**0.002**
(f) ≥ 22 W.A.	*ß*	CI-L	CI-U	P	boot. P
Brain weight	0.35	0.11	0.57	0.007	0.011
Genotype	**-0.57**	**-0.82**	**-0.34**	<0.001	**<0.001**

Table S16. Results of general linear models (LMs) describing the effects of total brain weight and SCA1 genotype on the volume of molecular layer of dentate gyrus (DG-ML) of hippocampus. (**a-d**) Age cohort-specific models (N = 8 WT and 10 SCA1 mice). (**e-f**) Models based on merged data (N = 17 WT and 19 SCA1 mice).

W.A. = weeks of age. *d.f.* = degrees of freedom. *ß* = standardized regression coefficient. CI = limits for 95% confidence intervals (CI-L: lower limit, CI-U: upper limit) based on BCa bootstrap. P = significance based on parametric approach. boot. P = significance based on percentile bootstrap.

(a) 10 W.A.	*ß*	CI-L	CI-U	P	boot. P
Brain weight	0.56	0.24	0.97	0.001	0.007
Genotype	**-0.4**	**-0.80**	**-0.02**	0.018	**0.025**
(b) 15 W.A.	*ß*	CI-L	CI-U	P	boot. P
Brain weight	0.45	0.04	0.85	0.025	0.024
Genotype	**-0.53**	**-0.86**	**-0.22**	0.006	**<0.001**
(c) 22 W.A.	*ß*	CI-L	CI-U	P	boot. P
Brain weight	0.47	0.22	0.75	0.003	0.01
Genotype	**-0.53**	**-0.8**	**-0.25**	<0.001	**<0.001**
(d) 32 W.A.	*ß*	CI-L	CI-U	P	boot. P
Brain weight	0.4	-0.06	0.68	0.062	0.078
Genotype	**-0.49**	**-0.9**	**-0.16**	0.019	**0.007**
(e) ≤ 15 W.A.	*ß*	CI-L	CI-U	P	boot. P
Brain weight	0.46	0.24	0.65	<0.001	<0.001
Genotype	**-0.51**	**-0.75**	**-0.28**	<0.001	**<0.001**
(f) ≥ 22 W.A.	*ß*	CI-L	CI-U	P	boot. P
Brain weight	0.46	0.2	0.65	<0.001	0.001
Genotype	**-0.49**	**-0.74**	**-0.26**	<0.001	**<0.001**

Table S17. Results of models describing association between relative (**a**) and absolute **(b)** volume of cerebellar molecular layer (Cb-ML) and given sensitive indicator, specifically for young SCA1 mice (age ≤15 weeks; 19 animals).

ß = standardized regression coefficient. CI = limits for 95% confidence intervals (CI-L: lower limit, CI-U: upper limit) based on BCa bootstrap. P = significance based on parametric approach. boot. P = significance based on percentile bootstrap. An unusual difference between the significances from parametric vs. bootstrapping approaches is caused by 1 outlying individual with very big cerebellum (Suppl. Data1, ID = „388; fully parametric approaches are very sensitive to outlying values in cases of small sample sizes whereas bootstrapping approach is relatively robust).

**a) Relative CB-ML**	*ß*	CI-L	CI-U	P	boot. P
OF thigmotaxis	-0.22	-0.78	0.21	0.38	0.29
OF distance	0.03	-0.66	0.43	0.92	0.94
Rotarod latency	0.2	-0.37	0.61	0.42	0.57
MWM non-moving	-0.02	-0.3	0.64	0.95	0.93
T-maze errors rate	0.31	-0.002	0.89	0.19	0.054
T-maze inflexibility	-0.27	-0.65	0.35	0.29	0.24
FST immobility	-0.15	-0.7	0.2	0.51	0.41
**b) Absolute CB-ML**	*ß*	CI-L	CI-U	P	boot. P
OF thigmotaxis	-0.46	-0.97	0.05	0.047	0.059
OF distance	0.51	-0.12	0.81	0.014	0.15
Rotarod latency	**0.72**	**0.15**	**0.94**	<0.001	**0.044**
MWM non-moving	-0.62	-0.93	0.12	0.005	0.18
T-maze errors rate	0.18	-0.5	0.61	0.47	0.33
T-maze inflexibility	-0.33	-0.66	0.37	0.14	0.21
FST immobility	-0.28	-0.81	0.59	0.28	0.5

Table S18. Results of models describing association between CA-SRLM volume and given sensitive indicator, specifically for young SCA1 mice (age ≤15 weeks; 19 animals). Shortages are the same as in Suppl. Table S17.

Model	*ß*	CI-L	CI-U	P	boot. P
OF thigmotaxis	-0.10	-0.60	0.30	0.66	0.60
OF distance	-0.1	-0.66	0.31	0.68	0.58
Rotarod latency	0.12	-0.33	0.66	0.63	0.72
MWM non-moving	-0.2	-0.37	0.27	0.36	0.18
T-maze errors rate	0.1	-0.42	0.43	0.66	0.55
T-maze inflexibility	-0.15	-0.61	0.38	0.54	0.54
FST immobility	0	-0.41	0.54	1	0.96

Table S19. Results of models describing association between brain weight and given sensitive indicator, specifically for young SCA1 mice (age ≤15 weeks; 30 animals). Shortages are the same as in Suppl. Table S17.

Model	*ß*	CI-L	CI-U	P	boot. P
OF thigmotaxis	-0.26	-0.57	0.16	0.62	0.13
OF distance	-0.24	-0.52	0.16	0.2	0.14
Rotarod latency	0.1	-0.2	0.59	0.61	0.60
MWM non-moving	-0.05	-0.45	0.42	0.79	0.80
T-maze errors rate	0.04	-0.34	0.34	0.84	0.80
T-maze inflexibility	-0.13	-0.49	0.22	0.50	0.47
FST immobility	-0.11	-0.47	0.42	0.57	0.63

Table S20. Results of models describing association between volume of DG-ML and given sensitive indicator, specifically for young SCA1 mice (age ≤15 weeks; 19 animals). Shortages are the same as in Suppl. Table S17.

Model	*ß*	CI-L	CI-U	P	boot. P
OF thigmotaxis	-0.31	-0.70	0.31	0.29	0.23
OF distance	-0.31	-0.55	0.49	0.38	0.40
Rotarod latency	0.27	-0.04	1.01	0.25	0.16
MWM non-moving	**-0.33**	**-0.96**	**-0.06**	0.12	**0.044**
T-maze errors rate	0.17	-0.21	0.49	0.46	0.35
**T-maze inflexibility**	**-0.48**	**-0.86**	**-0.03**	0.045	**0.019**
**FST immobility**	**-0.49**	**-0.74**	**-0.08**	0.024	**0.026**

Table S21. Results of models describing association between DG-ML volume and given sensitive indicator adjusted for effect of SCA1 genotype, specifically for young mice (age ≤15 weeks) of both genotypes (N = 17 WT and 19 SCA1 mice). Shortages are the same as in Suppl. Table S17.

(a) **OF thigmotaxis**	*ß*	CI-L	CI-U	P	boot. P
Genotype	0.27			0.21	
DG-ML volume	**-0.49**	**-0.89**	**-0.05**	0.025	**0.032**
(b) OF distance moved	*ß*	CI-L	CI-U	P	boot. P
Genotype	-0.58			0.02	
DG-ML volume	0.02	-0.35	0.47	0.93	0.94
(c) Rotarod latency	*ß*	CI-L	CI-U	P	boot. P
Genotype	-0.22			0.42	
DG-ML volume	0.15	-0.42	0.7	0.60	0.58
(d) **MWM non-moving**	*ß*	CI-L	CI-U	P	boot. P
Genotype	0.52			0.003	
DG-ML volume	**-0.39**	**-0.78**	**-0.13**	0.02	**0.005**
(e) T-maze errors	*ß*	CI-L	CI-U	P	boot. P
Genotype	0.83			<0.001	
DG-ML volume	0.14	-0.11	0.42	0.43	0.28
(f) T-maze inflexibility	*ß*	CI-L	CI-U	P	boot. P
Genotype	0.19			0.41	
DG-ML volume	-0.34	-0.76	0.09	0.16	0.10
(e) **FST immobility**	*ß*	CI-L	CI-U	P	boot. P
Genotype	0.21			0.26	
DG-ML volume	**-0.66**	**-1.04**	**-0.19**	0.001	**0.001**

Table S22. Results of models describing association between Cb-ML volume and given sensitive indicator adjusted for effect of SCA1 genotype, specifically for young mice (age ≤15 weeks) of both genotypes (N = 17 WT and 19 SCA1 mice). Shortages are the same as in Suppl. Table S17.

(a) OF thigmotaxis	*ß*	CI-L	CI-U	P	boot. P
Genotype	0.46			0.009	
Cb-ML volume	-0.24	-0.53	0.05	0.17	0.09
(b) OF distance moved	*ß*	CI-L	CI-U	P	boot. P
Genotype	-0.41			0.008	
Cb-ML volume	0.00	-0.35	0.29	0.99	0.98
(c) Rotarod latency	*ß*	CI-L	CI-U	P	boot. P
Genotype	-0.33			0.11	
Cb-ML volume	0.06	-0.40	0.45	0.78	0.84
(d) MWM non-moving	*ß*	CI-L	CI-U	P	boot. P
Genotype	0.67			<0.001	
Cb-ML volume	-0.12	-0.35	0.12	0.45	0.31
(e) T-maze errors	*ß*	CI-L	CI-U	P	boot. P
Genotype	0.84			<0.001	
Cb-ML volume	0.14	-0.05	0.42	0.29	0.18
(f) T-maze inflexibility	*ß*	CI-L	CI-U	P	boot. P
Genotype	0.35			0.049	
Cb-ML volume	-0.25	-0.51	0.12	0.17	0.13
(e) FST immobility	*ß*	CI-L	CI-U	P	boot. P
Genotype	0.47			0.004	
Cb-ML volume	-0.27	-0.63	0.02	0.09	0.07

Table S23. Results of models describing association between CA-SRLM volume and given sensitive indicator adjusted for effect of SCA1 genotype, specifically for young mice (age ≤15 weeks) of both genotypes (N = 17 WT and 19 SCA1 mice). Shortages are the same as in Suppl. Table S17.

(a) OF thigmotaxis	*ß*	CI-L	CI-U	P	boot. P
Genotype	0.50			0.012	
CA-SRLM volume	-0.21	-0.61	0.22	0.27	0.29
(b) OF distance moved	*ß*	CI-L	CI-U	P	boot. P
Genotype	-0.45			0.023	
CA-SRLM volume	0.22	-0.11	0.65	0.26	0.25
(c) Rotarod latency	*ß*	CI-L	CI-U	P	boot. P
Genotype	-0.31			0.2	
CA-SRLM volume	0.04	-0.45	0.52	0.85	0.87
(d) MWM non-moving	*ß*	CI-L	CI-U	P	boot. P
Genotype	0.73			<0.001	
CA-SRLM volume	-0.14	-0.36	0.13	0.36	0.24
(e) T-maze errors	*ß*	CI-L	CI-U	P	boot. P
Genotype	0.78			<0.001	
CA-SRLM volume	0.08	-0.20	0.31	0.59	0.48
(f) T-maze inflexibility	*ß*	CI-L	CI-U	P	boot. P
Genotype	0.36			0.083	
CA-SRLM volume	-0.13	-0.53	0.26	0.52	0.50
(e) FST immobility	*ß*	CI-L	CI-U	P	boot. P
Genotype	0.58			0.003	
CA-SRLM volume	-0.20	-0.53	0.18	0.28	0.23

Table S24. Results of models describing association between brain weight and given sensitive indicator adjusted for effect of SCA1 genotype, specifically for young mice (age ≤15 weeks) of both genotypes (N = 25 WT and 30 SCA1 mice). Shortages are the same as in Suppl. Table S17.

(a) OF thigmotaxis	*ß*	CI-L	CI-U	P	boot. P
Genotype	0.54			<0.001	
Brain weight	0.05	-0.31	0.27	0.87	0.87
(b) OF distance moved	*ß*	CI-L	CI-U	P	boot. P
Genotype	-0.50			<0.001	
Brain weight	-0.01	-0.31	0.34	0.91	0.92
(c) Rotarod latency	*ß*	CI-L	CI-U	P	boot. P
Genotype	-0.24			0.11	
Brain weight	0.09	-0.24	0.38	0.58	0.56
(d) MWM non-moving	*ß*	CI-L	CI-U	P	boot. P
Genotype	0.64			<0.001	
Brain weight	-0.02	-0.31	0.24	0.84	0.88
(e) T-maze errors	*ß*	CI-L	CI-U	P	boot. P
Genotype	0.72			<0.001	
Brain weight	-0.002	-0.19	0.19	0.99	0.97
(f) T-maze inflexibility	*ß*	CI-L	CI-U	P	boot. P
Genotype	0.48				
Brain weight	-0.09	-0.34	0.14	0.51	0.45
(e) FST immobility	*ß*	CI-L	CI-U	P	boot. P
Genotype	0.42			0.002	
Brain weight	-0.24	-0.47	0.05	0.073	0.080

Table S25. Results of linear mixed-effect models describing (partial) effect of the SCA1 genotype on density of $NeuN^+$ neurons (a, b) and NeuN immunofluorescence intensity (c, d) in CA1 (a, c) and CA2/3 (b, d) hippocampal pyramidal layer. The fluorescence intensity was subtracted by fluorescence of the background (see Suppl. Methods and Suppl. Fig. S5). Because adding a grouping factor (block) improved model fit (measured by BIC) in case of NeuN signal in CA2/3, it was included into model as a covariate. N = 5 mice per group with 4 evaluated sections per mouse.

ß = standardized partial regression coefficient. CI = limits for 95% confidence intervals of *ß* (CI-L: lower limit, CI-U: upper limit) based on percentile bootstrap. P = significance based on parametric approach. boot. P = significance based on percentile bootstrap. perm. P = significance based on permutation test of LME.

(a) Neuronal density – CA1	*ß*	CI-L	CI-U	P	boot. P	perm. P
Genotype	-0.28	-0.63	0.08	0.16	0.13	0.14
(b) Neuronal density – CA2/3	*ß*	CI-L	CI-U	P	boot. P	perm. P
Genotype	-0.38	-0.75	0.01	0.09	0.054	0.052
(c) **NeuN signal – CA1**	*ß*	CI-L	CI-U	P	boot. P	perm. P
Genotype	**-0.53**	**-0.86**	**-0.21**	0.013	**<0.001**	**0.002**
(d) **NeuN signal – CA2/3**	*ß*	CI-L	CI-U	P	boot. P	perm. P
Block				0.014		0.001
Genotype	**-0.58**	**-0.81**	-0.37	0.002	**<0.001**	**<0.001**

Table S26. Results of linear mixed-effect models describing the effect of the genotype on immunofluorescent markers related neurogenesis or neuroplasticity. The DCX^+ neuronal dendrites were quantified by means of the number of crossing lines (at least 200 µm per evaluated DG) located in three positions: i) the border between the DG granular and molecular layers (*M/G*); ii) the inner half of the DG-ML (closer to the granular layer; *M-in*) and iii) the outer half of the DG-ML (*M-out*). The PSA-NCAM immunofluorescent signal was measured in the DG hilus (DG-PL), DG-ML, CA4 pyramidal and CA1 stratum lacunosum-moleculare (CA1-SLM) layers (Suppl. Fig. S5). N = 5 mice per group with 4 measurement (i.e. evaluated hippocampi) per mouse. Other shortages are the same as in Suppl. Table S25. See Suppl. Methods for details.

	ß	CI-L	CI-U	P	boot. P	perm. P
**DCX^+PSA-$NCAM^+$ neurons**	**-0.67**	**-0.99**	**-0.35**	0.004	**<0.001**	**<0.001**
**DCX^+ dendrites (M/G)**	**-0.85**	**-1.08**	**-0.62**	<0.001	**<0.001**	**<0.001**
**DCX^+ dendrites (M-in)**	**-0.84**	**-1.08**	**-0.60**	<0.001	**<0.001**	**<0.001**
**DCX^+ dendrites (M-out)**	**-0.72**	**-1.02**	**-0.44**	0.001	**<0.001**	**<0.001**
PSA-NCAM signal (DG-PL)	-0.34	-0.88	0.21	0.26	0.23	0.21
PSA-NCAM signal (DG-ML)	-0.48	-0.92	0.05	0.12	0.074	0.069
PSA-NCAM signal (CA4-PL)	-0.36	-0.79	0.07	0.15	0.11	0.1
**PSA-NCAM signal (CA1-SLM)**	**-0.49**	**-0.83**	**-0.15**	0.022	**0.005**	**0.005**

Table S27. Results of linear mixed-effect models describing effect of SCA1 genotype on specific citrate synthase activity (log[mIU/mg of tissue]) and mitochondrial respiration (pmolO_2/s/mg) in cerebellar (**a**) and hippocampal (**b)** tissue. Following states of mitochondrial respiration were evaluated: 1) complex I OXPHOS capacity in the ADP-activated state of oxidative phosphorylation (*P I*). 2) Complex I + II OXPHOS capacity (*P I+II*). 3) Maximum capacity for electron transport (*E I+II*). 4) Complex II uncoupled capacity (*E II*). 5) *Complex IV* capacity. N = 5 WT and 6 SCA1 animals (in quadruplicates). Shortages are the same as in Suppl. Table S25

**(a)** Cerebellum	*ß*	CI-L	CI-U	P	boot. P	perm. P
Specific citrate synthase activity	0.04	-0.54	0.59	0.90	0.90	0.91
P I	-0.09	-0.60	0.42	0.72	0.78	0.72
P I+II	-0.06	-0.58	0.45	0.82	0.81	0.83
E I+II	-0.12	-0.62	0.41	0.67	0.66	0.67
E II	-0.02	-0.55	0.54	0.93	0.92	0.93
Complex IV	0.06	-0.47	0.59	0.83	0.83	0.82
**(b)** Hippocampus	*ß*	CI-L	CI-U	P	boot. P	perm. P
Specific citrate synthase activity	-0.49	-1.00	0.00	0.08	0.05	0.058
**P I**	**-0.62**	**-1.03**	**-0.20**	0.018	**0.004**	**0.007**
**P I+II**	**-0.63**	**-1.05**	**-0.21**	0.016	**0.003**	**0.005**
**E I+II**	**-0.65**	**-1.06**	**-0.25**	0.011	**0.002**	**0.003**
**E II**	**-0.67**	**-1.10**	**-0.23**	0.014	**0.002**	**0.006**
**Complex IV**	**-0.64**	**-1.09**	**-0.24**	0.013	**0.001**	**0.004**

Table S28. Results of linear mixed-effect models describing partial effects of SCA1 genotype and specific citrate synthase activity (mIU/mg) on mitochondrial respiration (pmolO_2/s/mg) in cerebellar (**a-e**) and hippocampal (**f-i)** tissue. Following states of mitochondrial respiration were evaluated: 1) complex I OXPHOS capacity in the ADP-activated state of oxidative phosphorylation (*P I*). 2) Complex I + II OXPHOS capacity (*P I+II*). 3) Maximum capacity for electron transport (*E I+II*). 4) Complex II uncoupled capacity (*E II*). 5) *Complex IV* capacity. N = 5 WT and 6 SCA1 animals (in quadruplicates).

Shortages are the same as in Suppl. Table S25

(a) Cb – P I	*ß*	CI-L	CI-U	P	boot. P	perm. P
Citrate synthase activity	0.64			<0.001		
Genotype	-0.14	-0.74	0.44	0.65	0.66	0.66
(b) Cb – P I + II	*ß*	CI-L	CI-U	P	boot. P	perm. P
Citrate synthase activity	0.67			<0.001		
Genotype	-0.11	-0.50	0.29	0.60	0.59	0.60
(c) Cb – E I + II	*ß*	CI-L	CI-U	P	boot. P	perm. P
Citrate synthase activity	0.72			<0.001		
Genotype	-0.17	-0.53	0.20	0.39	0.38	0.38
(d) Cb – E II	*ß*	CI-L	CI-U	P	boot. P	perm. P
Citrate synthase activity	0.72			<0.001		
Genotype	-0.07	-0.32	0.17	0.57	0.57	0.59
(e) Cb – complex IV	*ß*	CI-L	CI-U	P	boot. P	perm. P
Citrate synthase activity	0.70			<0.001		
Genotype	0.01	-0.28	0.30	0.93	0.93	0.93
(f) Hp – P I	*ß*	CI-L	CI-U	P	boot. P	perm. P
Citrate synthase activity	0.60			<0.001		
Genotype	-0.31	-0.69	0.06	0.14	0.10	0.12
(g) **Hp – P I + II**	*ß*	CI-L	CI-U	P	boot. P	perm. P
Citrate synthase activity	0.68			<0.001		
Genotype	**-0.29**	**-0.56**	**-0.01**	0.07	**0.039**	**0.048**
(h) **Hp – E I + II**	*ß*	CI-L	CI-U	P	boot. P	perm. P
Citrate synthase activity	0.73			<0.001		
Genotype	**-0.29**	**-0.48**	**-0.10**	0.015	**0.004**	**0.006**
(i) **Hp – E II**	*ß*	CI-L	CI-U	P	boot. P	perm. P
Citrate synthase activity	0.60			<0.001		
Genotype	**-0.36**	**-0.63**	**-0.08**	0.031	**0.010**	**0.022**
(j) **Hp – complex IV**	*ß*	CI-L	CI-U	P	boot. P	perm. P
Citrate synthase activity	0.60			<0.001		
Genotype	**-0.36**	**-0.62**	**-0.09**	0.025	**0.011**	**0.012**

References

1. Chaussenot, R. *et al.* Cognitive dysfunction in the dystrophin-deficient mouse model of Duchenne muscular dystrophy: A reappraisal from sensory to executive processes. *Neurobiol. Learn. Mem.* **124**, 111–122 (2015).
2. Darbra, S. & Pallarès, M. Alterations in neonatal neurosteroids affect exploration during adolescence and prepulse inhibition in adulthood. *Psychoneuroendocrinology* **35**, 525–535 (2010).
3. Guariglia, S. R. & Chadman, K. K. Water T-maze: A useful assay for determination of repetitive behaviors in mice. *J. Neurosci. Methods* **220**, 24–29 (2013).
4. Serchov, T., van Calker, D. & Biber, K. Sucrose Preference Test to Measure Anhedonic Behaviour in Mice. *BIO-PROTOCOL* **6**, (2016).
5. Basler, L., Gerdes, S., Wolfer, D. P., Slomianka, L. & Murray, K. D. Sampling the Mouse Hippocampal Dentate Gyrus. **11**, 1–10 (2017).
6. Kuznetsov, A. V. *et al.* Evaluation of mitochondrial respiratory function in small biopsies of liver. *Anal. Biochem.* **305**, 186–194 (2002).
7. Good, P. I. *Permutation, parametric and bootstrap tests of hypotheses*. (Springer, 2005).
8. Torchiano, M. Effsize - a package for efficient effect size computation. (2016). doi:10.5281/ZENODO.1480624
9. Canty, A. & Ripley, B. D. boot: Bootstrap R (S-Plus) Functions. (2017).
10. Pinheiro, J., Bates, D., DebRoy, S., Sarkar, D. & R Core Team. nlme: Linear and Nonlinear Mixed Effects Models. (2018).
11. Luo, D., Ganesh, S. & Koolaard, J. predictmeans: Calculate Predicted Means for Linear Models. (2018).
12. Benjamini, Y. & Hochberg, Y. Controlling the false discovery rate: a practical and powerful approach to multiple testing. *J. R. Stat. Soc. Ser. B* **57**, 289–300 (1995).
13. Oksanen, J. *et al. Package 'vegan' Title Community Ecology Package*. (2019).
14. Robin, X. *et al.* pROC: an open-source package for R and S+ to analyze and compare ROC curves. *BMC Bioinformatics* **12**, 77 (2011).
15. Diciccio, T. J. & Efron, B. *Bootstrap Confidence Intervals*. *Source: Statistical Science* **11**, (1996).
16. Bates, D., Mächler, M., Bolker, B. & Walker, S. Fitting Linear Mixed-effect Models Using **lme4**. *J. Stat. Softw.* **67**, (2015).
